# Evolutionary history of callose synthases in terrestrial plants with emphasis on proteins involved in male gametophyte development

**DOI:** 10.1371/journal.pone.0187331

**Published:** 2017-11-13

**Authors:** Lenka Záveská Drábková, David Honys

**Affiliations:** Laboratory of Pollen Biology, Institute of Experimental Botany, Academy of Sciences of the Czech Republic, Rozvojová 263, Praha 6, Czech Republic; Wuhan University, CHINA

## Abstract

Callose is a plant-specific polysaccharide (β-1,3-glucan) playing an important role in angiosperms in many developmental processes and responses to biotic and abiotic stresses. Callose is synthesised at the plasma membrane of plant cells by callose synthase (CalS) and, among others, represents the main polysaccharide in the callose wall surrounding the tetrads of developing microspores and in the growing pollen tube wall. CalS proteins involvement in spore development is a plesiomorphic feature of terrestrial plants, but very little is known about their evolutionary origin and relationships amongst the members of this protein family. We performed thorough comparative analyses of callose synthase family proteins from major plant lineages to determine their evolutionary history across the plant kingdom. A total of 1211 candidate CalS sequences were identified and compared amongst diverse taxonomic groups of plants, from bryophytes to angiosperms. Phylogenetic analyses identified six main clades of CalS proteins and suggested duplications during the evolution of specialised functions. Twelve family members had previously been identified in *Arabidopsis thaliana*. We focused on five CalS subfamilies directly linked to pollen function and found that proteins expressed in pollen evolved twice. CalS9/10 and CalS11/12 formed well-defined clades, whereas pollen-specific CalS5 was found within subfamilies that mostly did not express in mature pollen vegetative cell, although were found in sperm cells. Expression of five out of seven mature pollen-expressed CalS genes was affected by mutations in *bzip* transcription factors. Only three subfamilies, CalS5, CalS10, and CalS11, however, formed monophyletic, mostly conserved clades. The pairs CalS9/CalS10, CalS11/CalS12 and CalS3 may have diverged after angiosperms diversified from lycophytes and bryophytes. Our analysis of fully sequenced plant proteins identified new evolutionary lineages of callose synthase subfamilies and has established a basis for understanding their functional evolution in terrestrial plants.

## Introduction

The colonisation of land by the ancestors of embryophytes represents the most significant event in the evolutionary history of terrestrial plants. Many key innovations or adaptations have appeared. The multi-layered pollen wall evolved to protect free-existing male gametophytes against environmental stress, which became critical when plants first colonised the terrestrial environment 600–450 million years ago. Algae, however, do not have complex spore walls [1]. The spores of ‘lower’ spore-bearing plants and the pollen of ‘higher’ seed plants are considered homologous [2]. Genes implicated in pollen-wall development in angiosperms are also present in mosses and lycopsids, suggesting that they may be involved in spore-wall development in basal plants. The molecular genetics of spore/pollen development may thus be highly conserved, despite the large morphological, functional, and developmental differences between spores and pollen. The key innovation was the ability to generate sporopollenin, an extremely tough and resistant polymer protecting haploid spores [3]. The composition of the pollen and pollen tube wall is unique [4] and has varied physically and chemically in different seed plant lineages as well as in response to the environmental aspects of pollination [5]. Studies of numerous mutations affecting pollen wall synthesis [6–18] revealed the importance of pollen wall for the proper function of male gametophyte and the complex regulation of cell wall synthesis by sporophytic and gametophytic tissues [6, 11, 18]. Pollen walls begin to organise before meiosis when meiocytes become surrounded by callose secreted by a tapetum [19].

Callose is a polysaccharide (β-1,3-glucan) that plays a fundamental role in angiosperms in many developmental processes including plasmodesmata formation and cytokinesis as well as in plant responses to biotic and abiotic stresses [20]. Callose is also involved in several stages of development of male gametophytes [21]. The role of callose in cell-wall development in other plant groups is less well defined [2]; for example callose is currently believed to be absent in pteridophytes (excluding only one genus, *Selaginella*). Callose has been identified in some bryophytes [22] around the spore mother cell, but its link to cell-wall development, if any, is not well understood [2]. Callose is generally sparsely produced in plants, representing only 0.3–5% of the total cell-wall content (*Arabidopsis* and *Miscanthus* [23]).

Twelve callose synthase (*CalS* or glucan synthase-like (*GSL*)) genes have been identified in *Arabidopsis thaliana* and classified in one gene family [24–26]. Comprehensive view in organ specific callose synthesis was done in *Arabidopsis* [27] and in *Triticum aestivum* [28]. Callose synthases are usually classified in two major groups, one contributing to fertility and cell division: *CalS1* (*GSL6*), *CalS5* (*GSL2*), *CalS9* (*GSL10*), *CalS10* (*GSL8*) and *CalS11* (*GSL1*), and the second providing the reinforcement of structural cell walls: *CalS3* (*GSL12*), *CalS7* (*GSL7*), *CalS8* (GSL4), *CalS12* (*GSL5*). The function of *CalS2* (*GSL3*), *CalS4* (*GSL9*) and *CalS6* (*GSL11*) is still unknown [25, 29]. All twelve callose synthases were detected during pollen development (eFP Browser [30]), however only CalS5 represented putative pollen-specific protein being highly expressed in anthers including microspores and pollen, and its role in microgametogenesis was not compensated by other pollen-expressed CalS proteins [6]. CalS5 may have evolved as a key enzyme responsible for callose synthesis required for pollen development and pollen-tube growth in higher plants [31].

Five of the 12 callose synthase genes have been reported to have a function during microsporogenesis and microgametogenesis (*CalS5*, *CalS9*, *CalS10*, *CalS11*, and *CalS12*; Table 1). Meiocytes in developing *Arabidopsis* anther locules are temporarily packed prior to meiosis in a layer of callose to prevent cohesion and fusion and its dissolution results in the release of free microspores [32]. Callose deposition continues during the second meiotic cytokinesis and finally encloses individual microspores within tetrads [33]. The separation of tetrads into free microspores involves degeneration of the callose wall by tapetum-secreted 1,3-beta-glucanase (callase) [34], and the callose wall appears to act as a mould wherein the primexine provides a blueprint for the formation of the exine pattern on the mature pollen grain [6, 35]. Microspores are released after the callose wall degradation and undergo critical pollen mitosis I (PMI) to form immature bicellular pollen grains, where generative and vegetative cells are first separated by a callose cell wall [32]. The degradation of callose in tetrads leading to microspore release is critical for the synchronisation of subsequent microspore development, namely PMI, and is ensured by the extracellular secretion of callase to anther locules by the tapetum [36].

**Table 1:** Summary of callose synthase proteins their function, and location in the *Arabidopsis thaliana*. Proteins active in pollen are underlined.

Gene/Protein	AGI	Function	References
**CalS1 (GLS6)**	AT1G05570	forms a complex with a UDP-glucose transferase and is localized at the cell plate during cytokinesis	[21, 26]
		regulates plasmodesmal permeability under pathogen infection and mechanical stress	[37]
**CalS2 (GLS3)**	AT2G31960	unknown	[21, 25, 29]
**CalS3 (GLS12)**	AT5G13000	callose accumulation in plasmodesmata	[21, 38]
**CalS4 (GLS9)**	AT5G36870	expressed in primordia of branching adaxial buds, exact role unknown	[21, 25, 29]
**CalS5 (GLS2)**	AT2G13680	callose deposition in the wall and callose plugs of germinated pollen tubes	[6, 7, 21, 39]
		required for exine formation during microsporogenesis and therefore pollen viability	[6, 7, 40]
		prevents pollen degeneration early in development	[7]
**CalS6 (GLS11)**	AT3G59100	unknown	[21, 25, 29, 41]
**CalS7 (GLS7)**	AT1G06490	required for callose deposition at the sieve plate	[6, 17]
**CalS8 (GLS4)**	AT3G14570	regulates plasmodesmal permeability under pathogen infection and mechanical stress	[37]
**CalS9 (GLS10)**	AT3G07160	involved in the entry of microspores into mitosis	[21, 40, 42]
		important for the microspore asymmetric division	[40]
**CalS10 (GLS8)**	AT2G36850	involved in pollen development, namely in the entry of microspores into mitosis	[21, 40]
		required for callose biosynthesis at the cell plate	[43]
		involved in stomatal pattering and deposition at the plasmodesmata	[44]
**CalS11 (GLS1)**	AT4G04970	cell plate formation in sporophytic tissues	[10, 21, 25, 34, 45]
		prevents callose wall degradation in microspores early in development	[34]
		function in formation of callose wall that separates microspores within tetrads	[34]
**CalS12 (GLS5)**	AT4G03550	synthesis of callose wall that separates microspores within tetrads	[34]
		prevents callose wall degradation in microspores early in development	[21, 34, 45]
		important for exine formation and pollen wall pattering	[34]
		function in cell plate formation in sporophytic tissues	[21]

CalS5, CalS9, CalS10, CalS11, and CalS12 have been shown to be involved in pollen development at and around the mitotic stage. CalS5 participates in callose deposition in the pollen wall and in the plugs of growing pollen tubes [7]. It is also essential for the formation of the callose wall surrounding pollen mother cells [40]. Three distinct roles have been defined for CalS5 in pollen development: patterning the exine layer, forming callose in pollen tubes, and preventing pollen degeneration early in development [7]. CalS11 and CalS12 play at least partially redundant roles in pollen development [34] but are expressed at lower level than CalS5 [6]. CalS9 and CalS10 are also co-expressed in lower amounts than CalS5 [6]. They are responsible for the formation of the callose wall separating the microspores in tetrads and during pollen-grain maturation. CalS12 plays also an important role in stress-induced callose deposition [46]. CalS9 and CalS10 are independently required for an asymmetric microspore division and entry of microspores into mitosis [40]. CalS9 is required for the entry of microspores into the first and second mitotic divisions and may function in later pollen development after the free-microspore stage [42].

There are two types of callose, peripheral callose and interstitial callose, which are synthesised in the tetrad by different callose synthases. The peripheral callose of the tetrad is absent in the knockout mutant *cals5-2*, but the interstitial callose is present. This observation clearly indicates that CalS5 is responsible for the synthesis of peripheral callose [6]. Moreover, the main regulator of auxin signalling, auxin response factor 17 (ARF17), directly binds to the *CalS5* promoter to regulate its expression for callose synthesis [47]. *CalS11* and *CalS12* genes are responsible for the interstitial tetrad callose synthesis [34].

The evolutionary relationships amongst the orthologues of plant callose synthases in model plants are poorly known [21, 48, 39]. Here we analysed a large data set of 1211 amino acid sequences of callose synthase family members to classify all representatives available across angiosperms, gymnosperms, lycopods, and bryophytes to determine the associated evolutionary processes in the plant kingdom. We demonstrated the expression of callose synthase family members in *A*. *thaliana* and *Nicotiana tabacum*, with special emphasis on different pollen developmental stages.

## Materials and methods

### *A*. *thaliana* and *N*. *tabacum* gene expression data

The expression data for *A*. *thaliana* were obtained from eFP Browser [30] and from other publicly available resources as described previously [49, 50, 51]. Briefly, the transcriptome datasets were downloaded from the NASCArray microarray database through the AffyWatch service [52] and normalized using freely available dChip 1.3 software (http://www.dchip.org) to the median probe intensity level; model-based gene-expression values were based on the Perfect Match-only model [53, 54].

Total RNA microarray data for *N*. *tabacum* callose synthases were obtained from previously published datasets [55, 56] and [57]. Subcellular fractions related to callose synthases mRNA translation and storage (polysomes and RNA storage EPP particles [58]) were isolated by sucrose gradient centrifugation as described previously [59]. From both fractions, total RNA was isolated using RNeasy Plant Mini Kit (Qiagen) and used for the hybridization of Agilent 44K Tobacco Gene Expression Microarray as described in [59]. Again, the transcriptomic datasets were normalized using publicly available dChip 1.3 software and further analysed as described above and in [57].

### Searching transcriptomes and genomes for CalS homologues

CalS homologues were identified by BLASTP searches using *A*. *thaliana* CalS1-12 proteins from the TAIR database (https://www.arabidopsis.org/; Table 1) to query NCBI protein databases (http://www.ncbi.nlm.nih.gov/). The BLASTP searches used default parameters, adjusted to the lowest E-value. The duplicates from all searches were eliminated. The Phytozome version 11 database (https://phytozome.jgi.doe.gov) was next searched for callose synthase proteins not found by BLASTP. Finally, we conducted an iterative search of the UniProt database (http://www.uniprot.org/). We analysed all sequences independently of their annotations, with no prior assumptions.

### Sequence alignment

Amino acid sequences were aligned using the Clustal Omega algorithm [60] in the Mobyle platform [61], with homology detection by HMM–HMM comparisons [62]. Protein isoforms with the same length were also used, because the differential expression patterns producing protein isoforms from various tissues suggested that isoforms could have different biological functions *in vivo* [63].

### Phylogenetic reconstruction

Maximum likelihood (ML) topology searches were performed with MEGA 7 [64] based on the JTT matrix-based model. Initial tree(s) for the heuristic search were obtained by applying the neighbour-joining and BioNJ algorithms to a matrix of pairwise distances estimated using a JTT model and then selecting the topology with the best log likelihood value. A discrete gamma distribution was used to model differences in evolutionary rate amongst sites (five categories (+*G*, parameter = 0.9166)). The model of rate variation allowed some sites to be evolutionarily invariable ([+*I*], 0.2289% sites). The analyses included 1211 (ML analysis) and 655 (ML and rooted MP analyses) amino acid sequences. The final data set contained a total of 4150 and 3435 positions, respectively. Phylogenetic trees were constructed and modified with iTOL v3.4 [65]. A bootstrap analysis under the ML criterion was run in RAxML due to the size of the data set [66]. The analyses were run with optimised equilibrium frequencies using the GTR model of evolution for tree inference.

Maximum-parsimony (MP) analysis was conducted in MEGA 7 using the Tree-Bisection-Regrafting algorithm with the initial trees obtained by the random addition of sequences. The analysis included 655 amino acid sequences. The final data set contained a total of 4970 positions.

Timetrees for each callose synthase with function in pollen were computed in MEGA 7 [64] using estimated divergence times for all branching points in a tree applying an approach based on the relative-time (RelTime) method [67], which does not require assumptions for variations in lineage rate. Divergence times for all branching points in the topology were calculated using ML based on the JTT matrix-based model.

### Identification of domains and conserved amino acid sequence motifs

The Pfam Motif Library [68], NCBI DART [69], and MEME 4.11.2 [70] were used to analyse the conserved motifs of the selected proteins and the reference sequences. The MEME search was set to identify a maximum of 50 motifs for each protein with a wide sequence motif from 2 to 50 and total number of sites from 2 to 600. The number and arrangement of introns and exons were analysed using Gene Structure Display Server version 2.0 [71] by aligning the coding sequences with the genomic sequences.

## Results

### Analysis of 12 CalS homologues in *A*. *thaliana*

**Fig 1:**
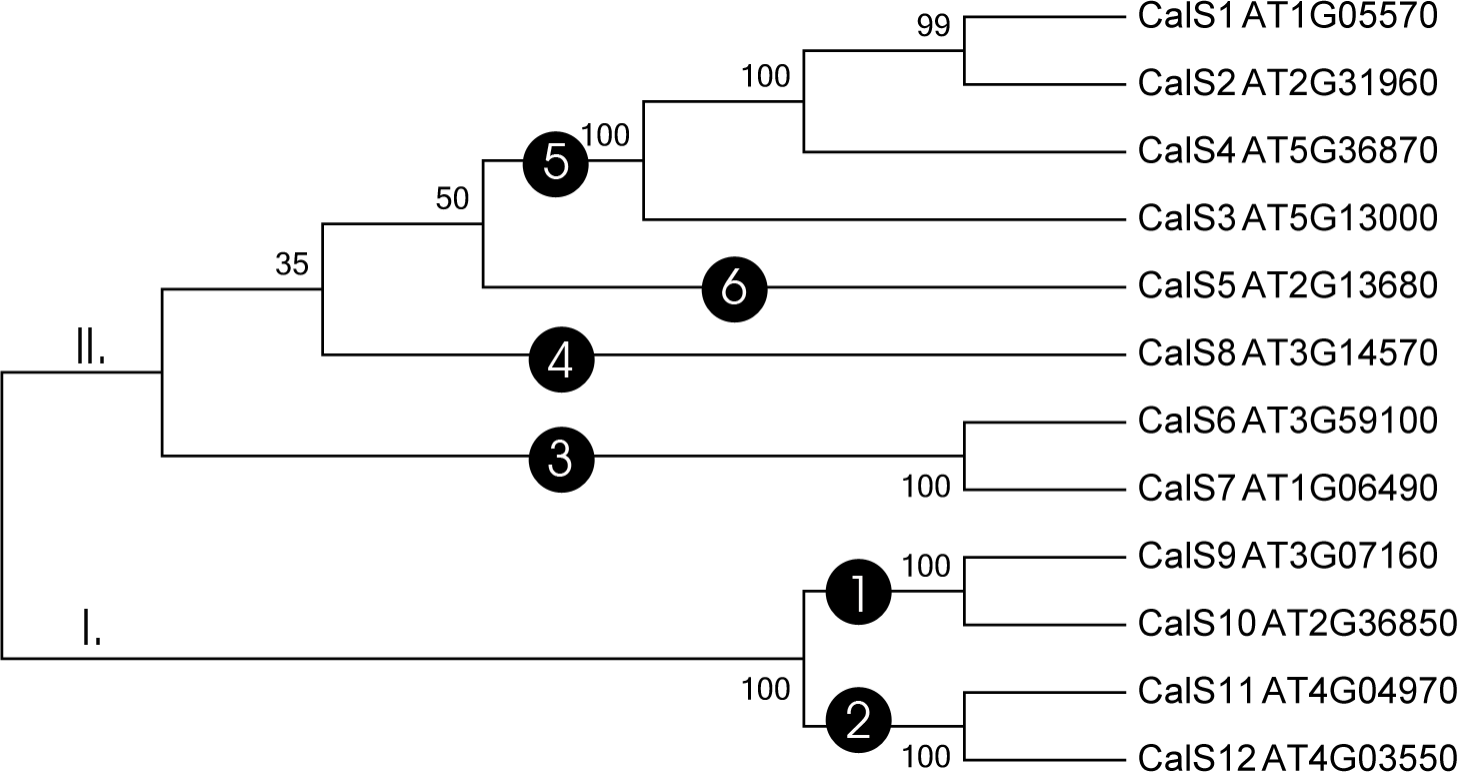
Phylogenetic tree of 12 *Arabidopsis thaliana* callose synthases. The evolutionary history was inferred using maximum likelihood. The bootstrap support values are shown next to the branches. Accessions are listed in Table 1.

The analysis of the 12 CalS homologues produced a phylogenetic tree depicting the relationships amongst all callose synthase family members in *A*. *thaliana* (Fig 1). The tree had two main groups, one containing CalS1-CalS8 and the second containing CalS9-CalS12. Subfamilies forming separate clades were CalS9/10 in clade 1, CalS11/12 in clade 2, CalS6 and CalS7 in clade 3, CalS8 in clade 4, CalS1, CalS2, CalS3 and CalS4 in clade 5, CalS5 in clade 6.

### Expression of callose synthase genes in different tissues of *A*. *thaliana* and in the pollen stages of *N*. *tabacum*

**Fig 2:**
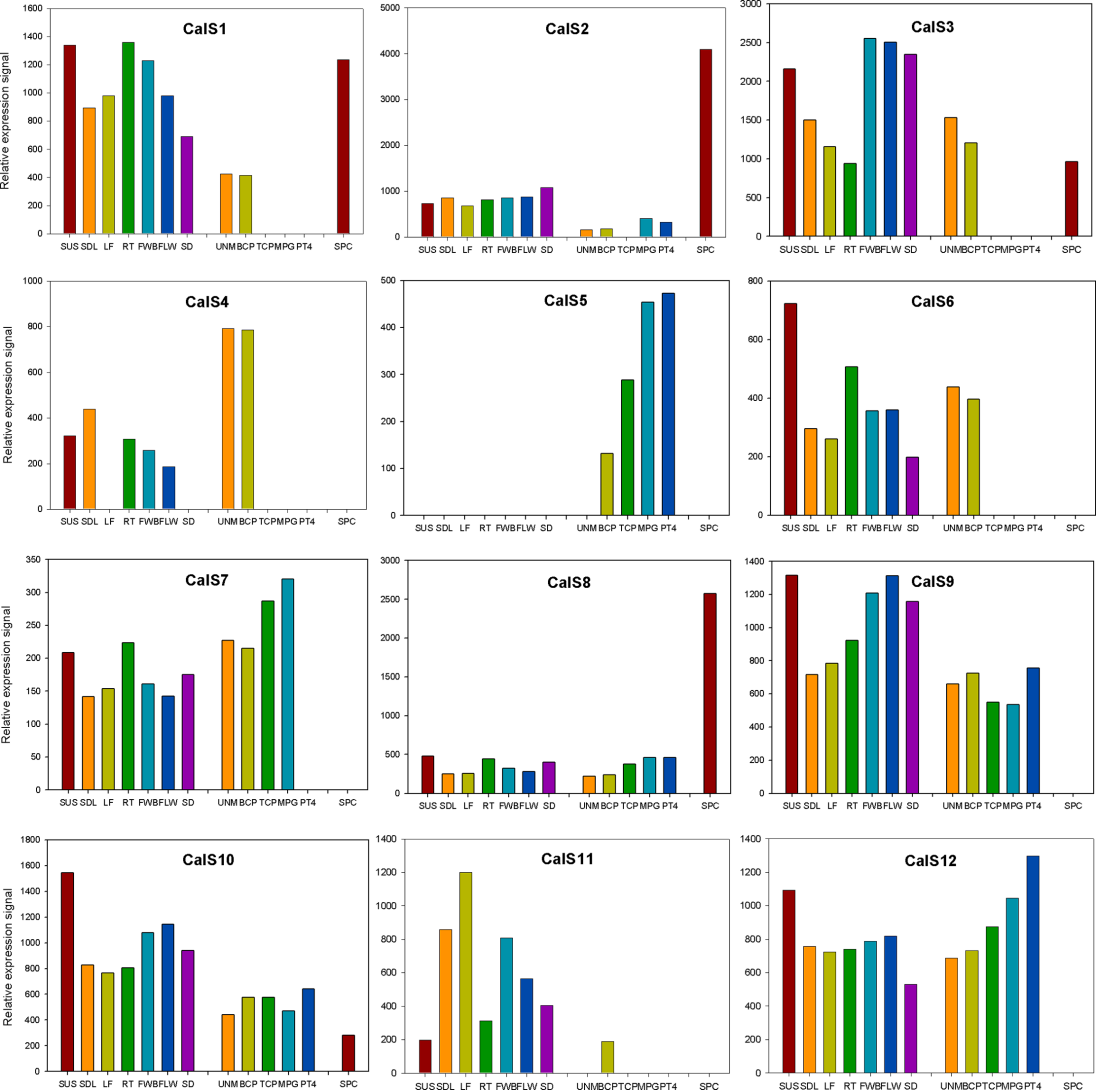
Expression profiles of 12 callose synthases in different sporophytic tissues and during male gametophyte development of *Arabidopsis thaliana*. SUS, suspension cultures; SDL, seedling; LF, leaf; RT, root; FWB, flower buds; FLW, flower; SD, seed; UNM, uninuclear microspore; BCP, bicellular pollen; TCP, tricellular pollen; MPG, mature pollen grain; PT4, 4h pollen tubes; SPC, sperm cells.

**Fig 3:**
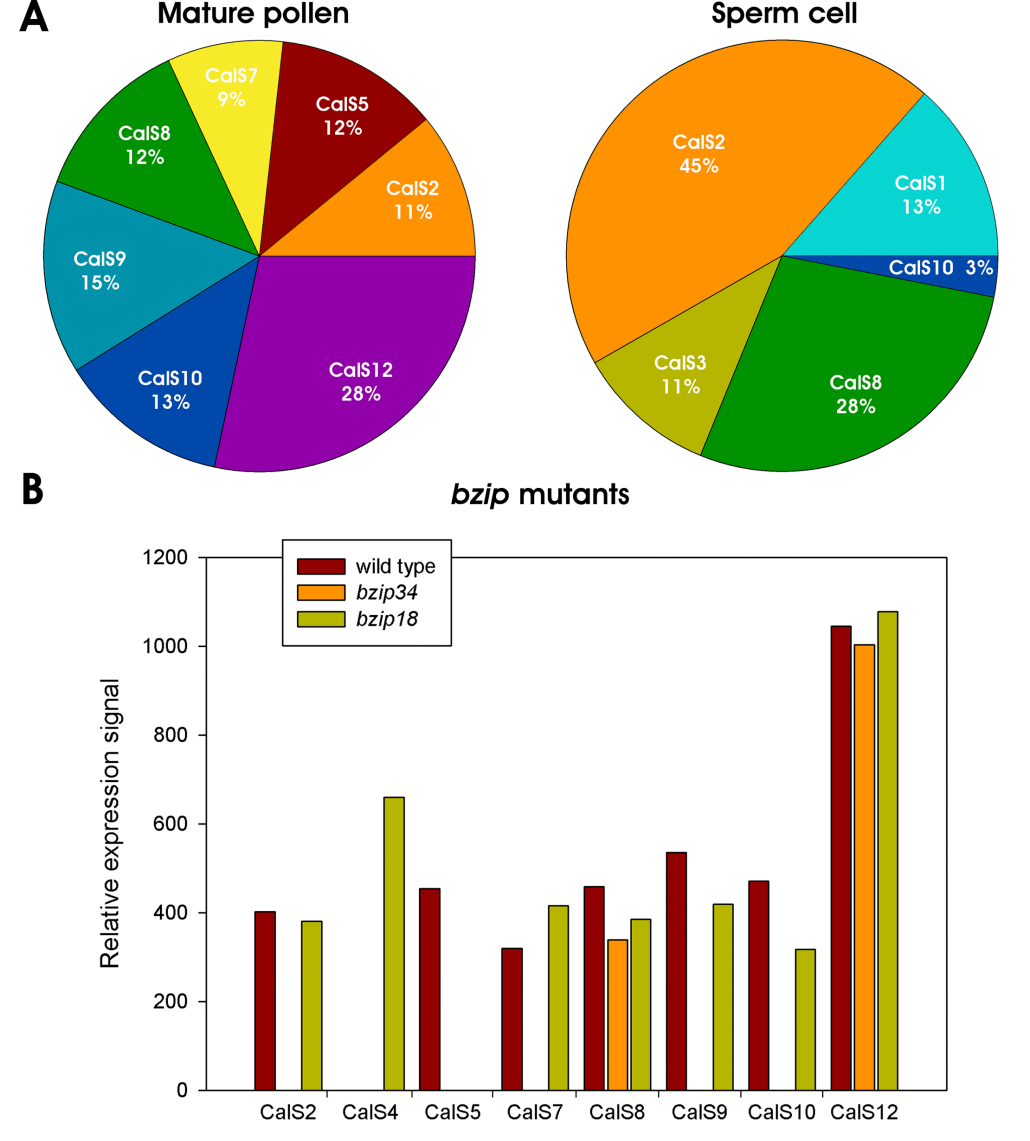
Callose synthase expression in *Arabidopsis thaliana* pollen. A) Abundance of expressed CalS transcripts in mature pollen and sperm cells. B) Expression profiles of pollen-expressed CalS transcripts in wild type Col-0 and *bzip18/-* and *bzip34/-* mutant *A*. *thaliana* mature pollen.

The callose synthase expression levels in different plant tissues, including male gametophyte, are shown in Fig 2. All callose synthase genes, except *CalS5* were expressed in most sporophytic tissues at various level. Similarly, detectable concentrations of all 12 callose synthases were found in at least several stages of *A*. *thaliana* pollen development [30, 49, 72]. However, pollen-expressed *CalS* genes showed three distinct expression patterns. The first group contained genes expressed only early in male gametophyte development that later disappeared or were expressed at very low level (*CalS1*, *CalS2*, *CalS3*, *CalS4* and *CalS6*). Based solely on the expression profile in the male gametophyte, *CalS11* can be put into this group too, however other characteristics (see below) associates this gene more likely with the group two. The second group comprised callose synthases that were expressed throughout male gametophyte development at higher abundance, often being accumulated in later developmental stages (CalS7, CalS8, CalS9, CalS10 and CalS12). All these proteins were expressed also in sporophytic tissues. The last expression group contained only one member, CalS5, that also accumulated throughout pollen development and progamic phase but unlike others, was pollen-specific. Finally, specific set of genes, partially overlapping with groups one and two, then comprised callose synthases that were active in male gametes, sperm cells (CalS1, CalS2, CalS3, CalS8 and CalS10). Although all these genes were expressed also in the sporophyte, they differed in their expression patterns throughout pollen development (Fig 2). Interestingly, sperm-cell expression of most of them was rather strong. Taken together, the most abundantly expressed callose synthase proteins in *A*. *thaliana* mature pollen were CalS12, CalS9 and CalS10, whereas sperm cells expressed mostly CalS2, CalS8 and CalS1 (Fig 3A).

bZIP family transcription factors bZIP18 and bZIP34 were previously shown to be involved also in the regulation of pollen wall formation [11, 18]. Transcriptomic analyses of pollen deficient in these proteins showed that many mature pollen-expressed callose synthases are in the regulons of either bZIP18, bZIP34 or both. Of seven callose synthases expressed in mature pollen, only two (CalS8 and CalS12) were not affected in *bzip18* or *bzip34* mutants (Fig 3B) suggesting that the regulation of their expression was independent of the activity of studied bZIP proteins. On the contrary, *CalS2*, *CalS7*, *CalS9* and *CalS10* expression was strongly downregulated in *bzip34* mutant pollen. The most severely affected was pollen-specific gene *CalS5*, that was downregulated in both *bzip18* and *bzip34* mutants independently. Finally, CalS4 that is usually expressed only during early male gametophyte development, was activated also in mature pollen in *bzip18* mutant pollen. Altogether, our data suggest that pollen callose synthases may be at least partly regulated by bZIP family transcription factors together with other proteins involved in the synthesis of cell wall formation [11, 18].

**Fig 4:**
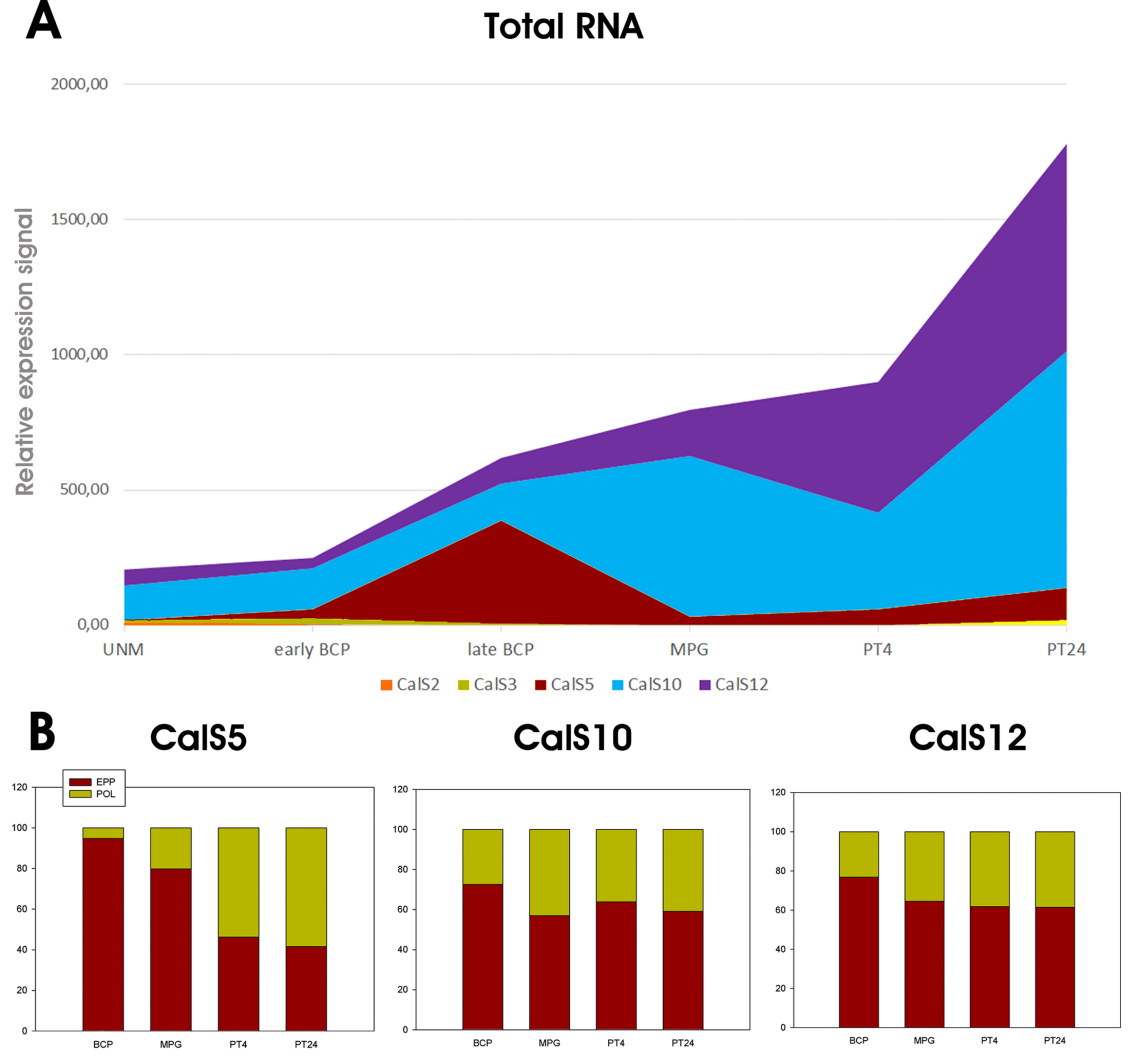
Callose synthase expression profiles in *Nicotiana tabacum* male gametophyte. A) Expression profile of five male gametophyte-expressed CalS mRNAs during pollen development and progamic phase, B) Distribution of transcripts encoding three most abundant tobacco pollen CalS mRNAs (CalS5, CalS10, CalS12) between actively translated polysomal and storage EPP fractions. UNM, uninuclear microspore; early BCP, early bicellular pollen; late BCP, late bicellular pollen; MPG, mature pollen grain; PT4, 4h pollen tubes; PT24, 24h pollen tubes; EPP, mRNA storage EPP complexes; POL, polysomal fraction.

Eight callose synthase subfamilies were found to be encoded in *N*. *tabacum* (CalS2, CalS3, CalS5, CalS7, CalS8, CalS10, CalS11 and CalS12). Of them, only five were expressed during pollen development (Fig 4), whereas remaining three were much more abundant. CalS5 abundance peaked at late bicellular stage before pollen maturation, whereas CalS10 and CalS12 were most strongly expressed after pollen germination, in growing pollen tubes (Fig 4A). Interestingly, the overall quantity of transcripts of three most abundant callose synthase transcripts was not reflected by their transitional efficiency [58, 73]. Whereas CalS10 and CalS12, peaking during progamic phase, were similarly distributed between storage EPP complexes and actively translating polysomes throughout the whole pollen development, CalS5 was more intensely translated after pollen germination when more callose synthase activity was also needed for pollen tube wall synthesis (Fig 4B).

### Identification and phylogenetic analysis of the CalS family in plants

**Fig 5:**
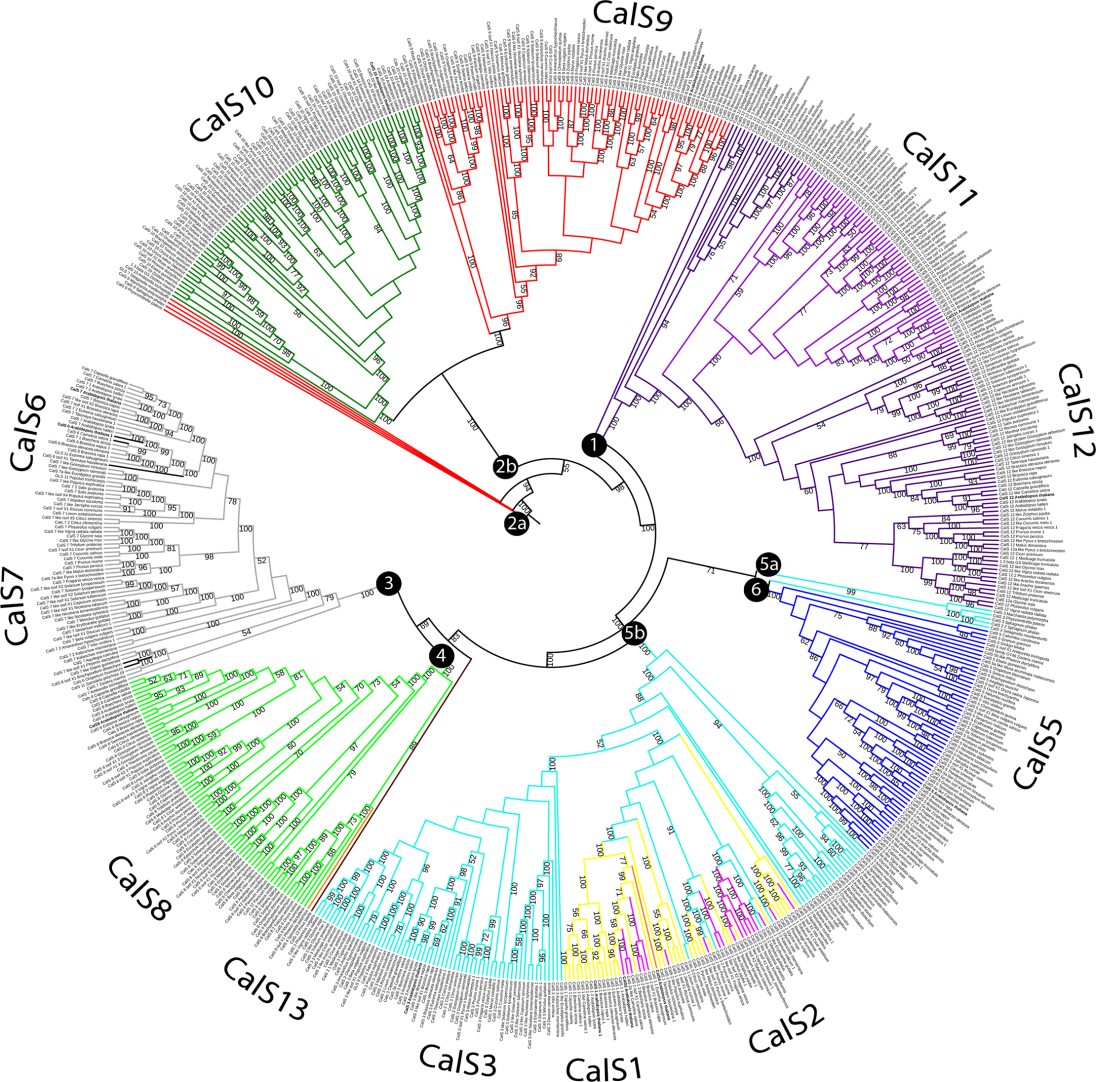
Unrooted phylogenetic tree of 655 proteins sequences of the callose synthase. The evolutionary history was inferred using maximum likelihood. Bootstrap support values ≥50% are shown on the branch above. The ML log likelihood -482640.553247. The analysis included 655 amino acid sequences and 3435 positions in the final data set. Numbers in circles indicate main clades described in the text.

**Fig 6:**
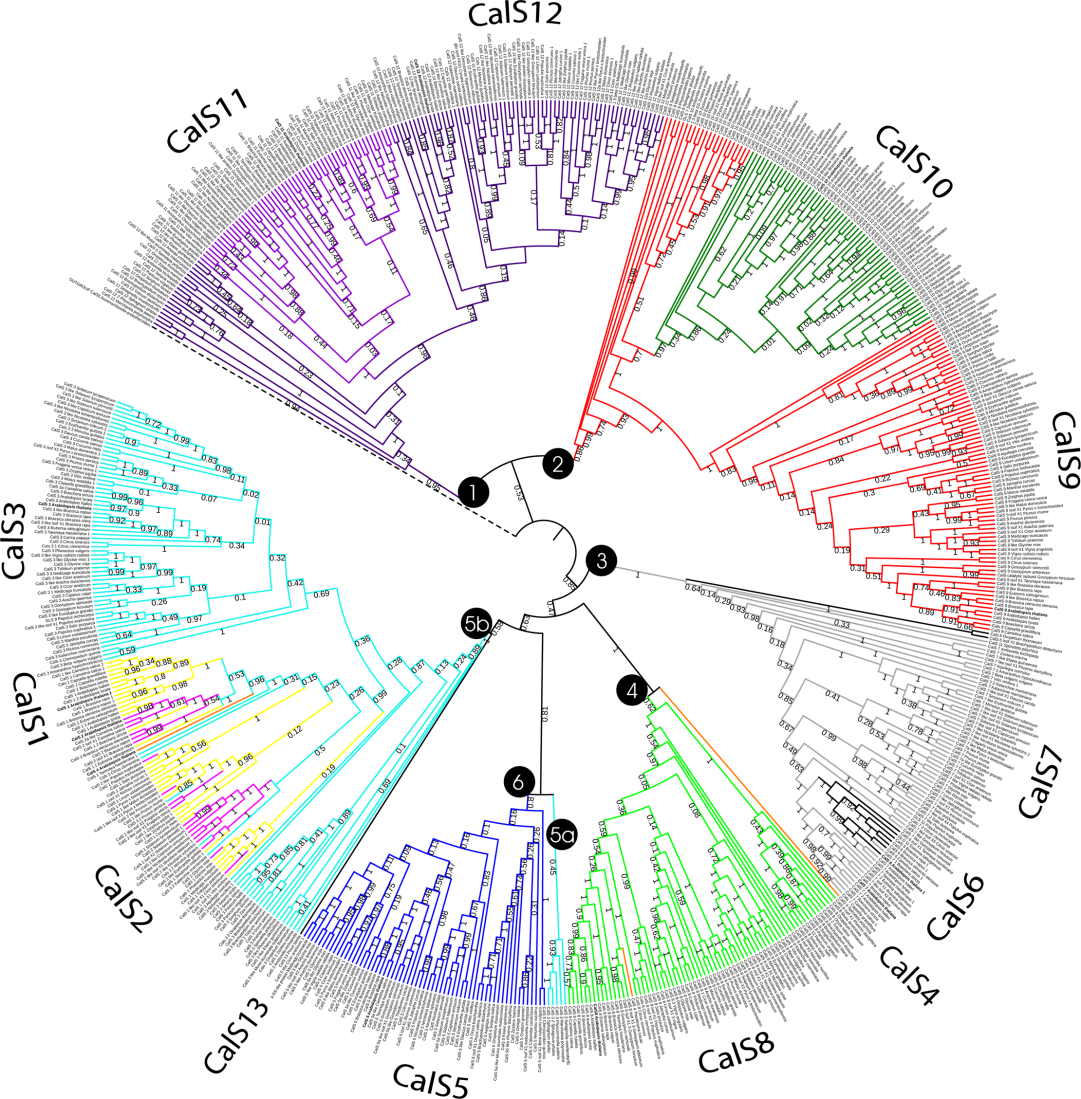
Callose synthase phylogeny in terrestrial plants inferred using maximum parsimony. **The tree is rooted by green alga *Auxenochlorella protothecoides* representing one of possible outgroup for terrestrial plants.** The most parsimonious tree with length 90012, CI = 0,223978, RI = 0,828990 is shown. The data contains 655 protein sequences and 4970 positions in the final dataset. Bootstrap support values ≥50% are shown next to the branches. Numbers in circles indicate main clades described in the text.

The 1211 sequences assembled from 101 plant species were analysed for the presence of CalS and CalS-like proteins. The evolutionary relationships amongst the callose synthases were determined using ML and MP analyses based on multiple alignments of the CalS proteins. The evolutionary hypotheses from these analyses were highly congruent. Most of the CalS proteins in the phylogenetic tree containing 1211 protein sequences formed main clades corresponding to the distribution of the 12 family members previously found in *A*. *thaliana* (Fig 1 and S1 Fig). Not all species contained orthologues of all 12 proteins; the number varied from four to twelve. Only a few species contained more than single-copy paralogues of a particular protein. Five CalS proteins directly linked to pollen function evolved twice; CalS9, 10, 11, and 12 formed a well-defined clade (clades 1 and 2), and the most prominent pollen-specific protein, CalS5 (clade 6), was embedded inside the subfamilies in the MP tree that were expressed in pollen at lower lever (Figs 5 and 6 and S1 Fig). Therefore, CalS5 formed a sister group to the pollen non specifically-expressed group comprising CalS3/2/1, CalS8, CalS7, and CalS6 in the ML tree (clades 3, 4 and 5; Fig 5 and S1 Fig). Only three subfamilies, CalS5, CalS9, and CalS11, however, formed monophyletic, conserved clades. Similar situation was found for other model plant with sequenced genome, *Nicotiana tabacum*, allotetraploid species that arose from the hybridization of *N*. *sylvestris* and *N*. *tomentosiformis* [74]. Only nine CalS proteins were found in *Nicotiana*, CalS1 from clade 5, CalS4 (clade 4) and CalS6 (clade 6) are missing. Members from each phylogenetic group (order) tended to cluster together within a given subfamily represented by a clade, suggesting that different callose synthase subfamilies could have expanded after the divergence from their common ancestor to form their own subclasses within each CalS subfamily (e.g. bryophytes, lycophytes, monocots, and dicots).

CalS9 formed two distinct clades (Figs 5 and 6 and S1 Fig), the first formed in angiosperms and the second grouping bryophytes (*Physcomitrella* and *Sphagnum*) and lycopods (*Selaginella*). CalS10 formed monophyletic clade inside CalS9. The CalS11 clade was monophyletic but embedded inside CalS12. CalS6 formed a separate clade mixed with CalS7 (clade 3). The CalS7 subfamily contained the basal angiosperm *Amborella trichopoda* in the basal part of the clade. CalS3 was divided into three groups, one found in bryophytes (*Physcomitrella* and *Sphagnum*) and lycopods (*Selaginella*) in the clade 5a and basal for CalS5, one present mostly monocots and basal for CalS1 and CalS2, and the third CalS3 clade grouping only dicots, which clustered together with the second group. The CalS8 subfamily formed a separate clade 4, but contained *Spirodela polyrhiza* CalS4 as a sister group and embedded *Theobroma cacao* CalS4 inside the CalS8 subfamily.

Only one representative from gymnosperms, the CalS13 protein from *Pinus taeda*, was supported in a basal position of the CalS3 clade. The sequence from *P*. *taeda* was used, although it was about 50% shorter than the sequences in the rest of the matrix, because only the most informative sites were included. Basal angiosperms from the study [39] were similarly retained in the analyses.

We next determined the divergence of the time estimates for all branching points in the callose synthase trees associated with pollen development. Relative-timed ML phylogenetic trees established for the callose synthase subfamilies involving pollen (CalS5, CalS9, CalS10, CalS11, and CalS12) strongly supported closer relationships within the main taxonomic groups (orders, S2–S6 Figs). Branch lengths indicated the length of time between nodes on a relative timescale. Relative time within each branch did not differ substantially, indicating a common origin and higher conservation in the callose synthase proteins. Only the relative times for bryophytes and lycopods diverged greatly, similarly to the evolutionary distance from angiosperms (S3 and S6 Figs). These results suggested relatively short evolutionary time of divergence within each subfamily.

### The distribution of conserved amino acid domains and motifs within the CalS clades

The comparison of the sequences of the callose synthase subfamilies indicated that members of the same subfamily were closely related to each other and distantly related to the members of other subfamilies, consistent with the results of the phylogenetic analyses. The 12 *CalS* genes in *A*. *thaliana* were distributed over the five chromosomes [26]: chromosome 1, *GLS6* (*CalS1*) and *GLS7* (*CalS7*); chromosome 2, *GLS2* (*CalS5*), *GLS3* (*CalS2*), *GLS8* (*CalS10*) and *GLS11* (*CalS6*); chromosome 3, *GLS4* (*CalS8*) and *GLS10* (*CalS9*); chromosome 4, *GLS1* (*CalS11*) and *GLS9* (*CalS4*), *GLS5* (*CalS12*); and chromosome 5, Chromosome 5 and *GLS12* (*CalS3*). The genes expressed abundantly in pollen were on chromosomes 3–5 (Table 1).

**Fig 7:**
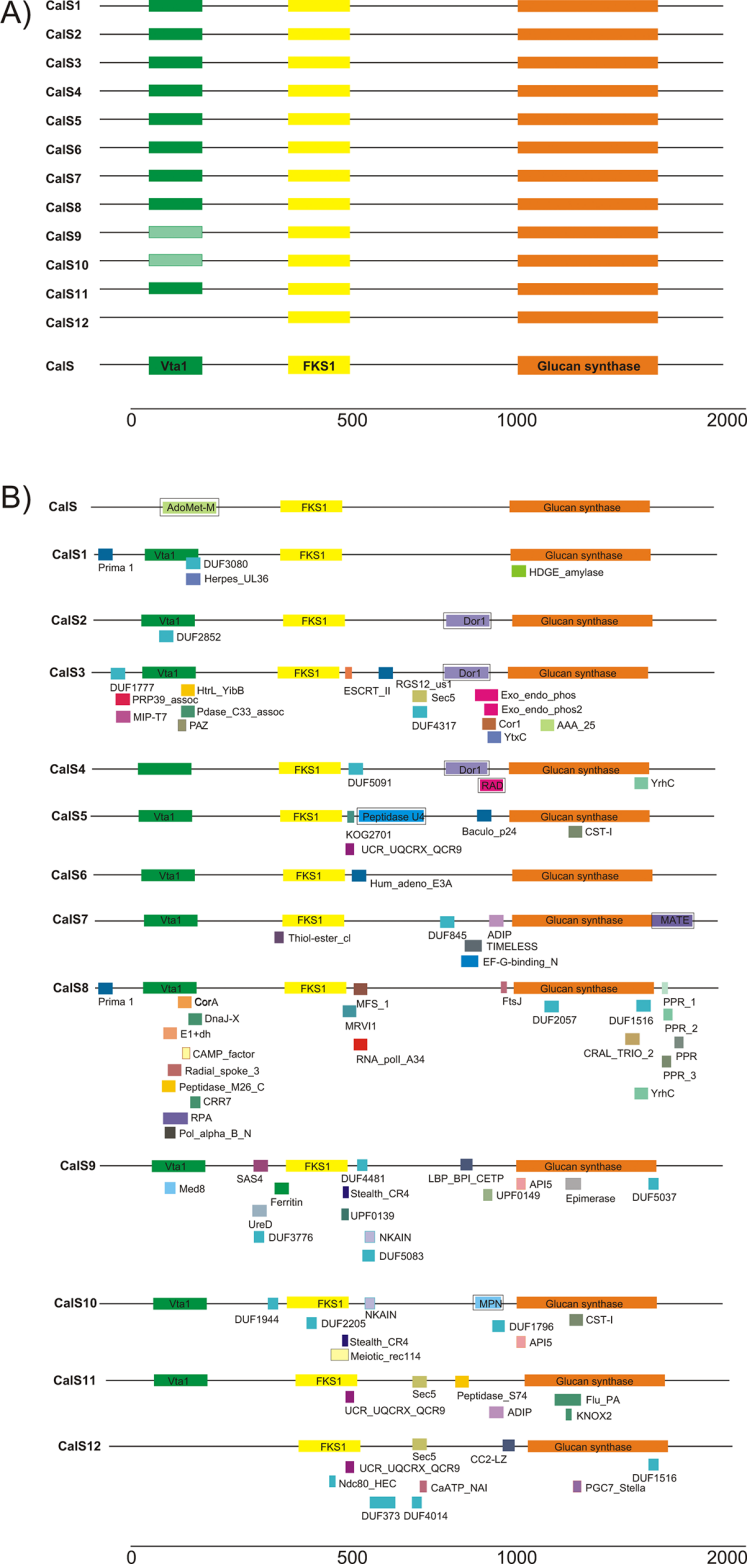
Consensus domains in the callose synthase proteins. A) Three main domains from the Vta1 superfamily, 1,3-beta-glucan synthase subunit FKS1, and the 1,3-beta-glucan synthase superfamily. B) Specific domains for each callose synthase. Domains were predicted by Pfam, and the domain in the boxes was predicted by DART. See S1 and S2 Tables for details.

Most of the *CalS* genes encoded proteins of about 2000 amino acids in length. CalS proteins typically consisted of three conserved domains: Vta1, FKS1, and glucan synthase (Fig 7). The Vta1 domain was present in all subfamilies but sometimes was not found in CalS9 and Cals10 proteins. Vta1 is involved in the transport of the multivesicular bodies, an endosomal compartment involved in sorting membrane proteins for degradation in lysosomes [75]. This division fully corresponded to the distribution of the CalS subfamilies, where CalS9 and Cals10 formed one well-supported clade (Figs 4 and 5). The FKS1 domain was highly conserved across the CalS proteins of terrestrial plants and was homologous with fungal FKS genes encoding an integral membrane protein, a subunit of 1,3-beta-D-glucan synthase [26, 76]. Further comparative analysis of CalS proteins in Pfam identified 81 types of protein domains (Fig 7). Some of them were unique for particular subfamilies (e.g. DUF3080 in CalS1 or KNOX2 in CalS11), and some were shared amongst two or more subfamilies. For example, UCR_UQCRX_QCR9 (ubiquinol-cytochrome C reductase) domains were shared amongst CalS5, CalS11, and CalS12, whereas API5 (apoptosis inhibitory protein) was shared by CalS9 and CalS10, and NKAIN (Na, K-ATPase interacting protein) was found in CalS9 and CalS11. S1 Table provides detailed information of the type, hypothetic function, and distribution of each domain within the species. Most of the domains were found in CalS8 (22) and CalS3 (16), and only one was found in CalS6. Callose synthases involved in the development of male gametophytes usually contained fewer domains (five in CalS5, six in CalS11, and nine in CalS10 and CalS12). To sum up, we found highly variable multidomain structure of callose synthase proteins within plants. It is generally believed that multidomain proteins evolved under selective pressure during evolution to create new function. From this point of view, CalS8, CalS3 and CalS9 seem to be under the highest evolutionary pressure. Moreover, in different phylogenetic groups of plants different domains evolved in response to different physiological and developmental signals. The detection of the MEME motifs indicated other structural diversification of the callose synthase proteins. MEME works by searching for repeated, ungapped sequence patterns that occur in the protein sequences. A set of proteins that interact with a single host protein may do so via similar domains. The details of the 50 putative motifs identified are shown in S2 Table. 44 motifs were conserved across all callose synthase subfamilies, and six motifs were more specific for two or more subfamilies. CalS1/2/3 and CalS5 contained motif 42, corresponding to clades 5 and 6 (Figs 5 and 6). CalS9/10 contained motif 50 that was specific to clade 6. Interestingly, CalS8/11/12 did not contain motif 14, Cal6/8 did not contain motif 23, CalS6/8/10/11/12 did not contain motif 32, and CalS6/10/11/12 did not contain motif 47. The functions of each motif were identified by searching Pfam, showing that nine motifs encode glucan synthase domain, two motifs belong to the FKS1 domain, five motifs belong to transmembrane domains, while the remaining 34 motifs do not appear to be associated with any domain.

**Fig 8:**
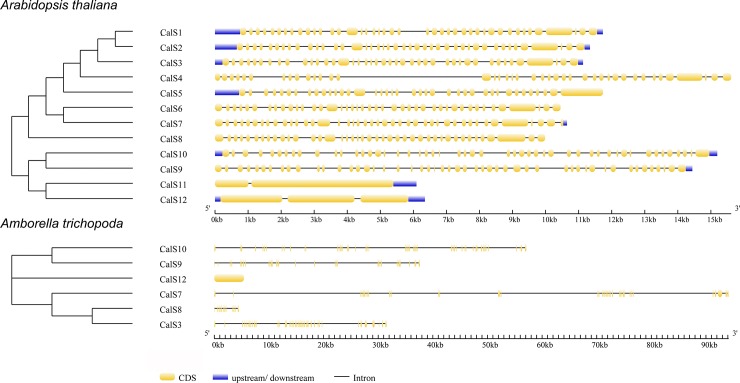
Comparison of the number and arrangement of introns and exons for representative species from a derived angiosperm (*Arabidopsis thaliana*) and a basal angiosperm (*Amborella trichopoda*).

Callose synthase genes showed remarkably complex exon/intron structure and with the exception of CalS11 and CalS12, they usually contained over 40 exons (Fig 8). For example, CalS1 gene comprised 42 exons and 41 introns and was transcribed into a 6-kb mRNA [25]. Similar complex arrangement was found also basal angiosperm (*A*. *trichopoda*) that highlighted that this feature is characteristic for all angiosperm phylogenetic group (Fig 8).

## Discussion

### Expression patterns of members of the callose synthase family

Genome analyses indicated that *A*. *thaliana* contained all 12 members of the callose synthase family. *N*. *tabacum*, however, contained only eight callose synthases, and three callose synthases abundantly expressed in pollen were distributed significantly differently during pollen development. CalS9, CalS10 and CalS12 were expressed at similar or higher levels than CalS5 in *A*. *thaliana*, although the non-redundant function for male gametophyte development was demonstrated only for pollen-specific CalS5 [6]. However, CalS5 expression in *N*. *tabacum* was lower than that of CalS10 and CalS12 in total pollen and this trend was even more apparent during the progamic phase. On the contrary, the putative translation activity of CalS5 transcript was higher than that of CalS10 and CalS12 in growing pollen tubes (Fig 4), This gradual translational activation in growing pollen tubes showed similar pattern as the translation of pollen-specific gene *ntp303* encoding pollen tube-wall glycoprotein p69 [58, 73].

CalS9/CalS10 and CalS11/CalS12 were closely related. The CalS10 clade was embedded within CalS9 in the phylogenetic tree, and CalS11 was similarly embedded within the CalS12 clade. The above results supported the suggestion in [42] that these members of the CalS family acted in a complex, or more likely, that these two pairs played at least partially redundant roles in pollen development [34]. In Arabidopsis, such hypothesis was supported only for co-expressed proteins CalS9 and CalS10, whereas the expression profiles of CalS11 and CalS12 were different; CalS12 was the most abundant late pollen-expressed callose synthase but CalS11 expression was much lower and at earlier developmental stages. It seemed that in *N*. *tabacum* pollen development, only CalS10 and CalS12 from these pairs were active. CalS10 and CalS12 may have specific developmental tasks in species with bicellular pollen, such as *N*. *tabacum*. Both members of these pairs, though, still may act in pollen development in species shedding tricellular pollen, such as *A*. *thaliana*. We can thus speculate which state is ancestral and which derived. In [77] proposed that bicellular pollen evolved secondarily from tricellular ancestors during shifts away from a rapid life cycle or from limited reproduction. We can follow up this hypothesis that during the evolution of derived species with bicellular pollen, reduction of possible redundant callose family members occurred and their role in pollen development was played by only one partner, i.e. CalS10 and CalS12 in this case. Interestingly, genes encoding eight CalS family members were identified in tobacco genome, however not all of them were expressed. Therefore, they might represent pseudogenes or might be silenced by the activity of matching miRNAs.

The absence of CalS6, CalS7 and CalS8 in tobacco pollen is similar to findings in [26]. They speculated that the absence of these callose synthases in EST database (based on leaf samples) suggested that these genes might be expressed at very low levels or that their expression pattern might be induced special conditions such as pathogen infection. However, since we found pollen-abundant CalS5 expressed also in *N*. *tabacum* [57]; we did not identify CalS6 and we can also assume that the expression of CalS7 and CalS8 is very limited in wild type pollen.

### Phylogenetic reconstruction and evolutionary history of CalS in terrestrial plants

Callose is synthesised by callose synthases encoded by members of the *CalS* gene family, which vary amongst terrestrial plants. *A*. *thaliana* contains 12 members in two main clades [21]. The number of CalS orthologues, however, varies within individual groups of terrestrial plants. Callose synthase subfamilies are structurally similar and likely diverged from a common ancestral glucan synthase domain, which is conserved in all subfamilies. Multiple *CalS* genes may have evolved in higher plants to catalyse callose synthesis in different locations and in response to different physiological and developmental signals [26]. Our phylogenetic analyses were designed to identify the relationships within the callose synthases subfamilies in detail.

In contrast to [6], we found two main groups and six subgroups in the phylogenetic tree of *A*. *thaliana* callose synthases. In [21] described four main subfamilies but did not include the support values for the tree branches. They also reported only a composite tree using ClustalW2 for phylogenetic analysis. According [21], the first subfamily contained CalS11 (AtGSL1), CalS12 (AtGSL5), CalS10 (AtGSL8), and CalS9 (AtGSL10), the second subfamily contained CalS5 (AtGSL2), CalS2 (AtGSL3), CalS1 (AtGSL6), and CalS3 (AtGSL12), the third subfamily comprised CalS7 (AtGSL7) and CalS6 (AtGSL11), and the fourth subfamily contained CalS8 (AtGSL4). The descriptions of the subfamilies, however, is unusual and lacking appropriate experimental support, and CalS4 (AtGLS9) was not included in any group. We analysed the same set of samples (Fig 1), generated an ML tree, and proposed the same principal two groups containing six subfamilies: subfamily 1, CalS9 and CalS10; subfamily 2, CalS11 and CalS12; subfamily 3, CalS6 and CalS7; subfamily 4, CalS8; subfamily 5, CalS1, CalS2 and CalS3; and subfamily 6, CalS5.

Our comprehensive phylogenetic analysis of the CalS family included 1211 and 655 sequences, respectively (Figs 5 and 6 and S1 Fig) from 101 plant species. The sequences grouped into six distinct clades in the phylogenetic tree (S1 Fig). Early events in the diversification of embryophytes gave rise to mosses, liverworts, and hornworts. All bryophyte lineages share a life cycle in which the gametophyte (haploid phase) dominates, with a sporophyte (diploid phase) dependent on the maternal gametophyte. Vascular plants instead have a dominant sporophytic phase and more or less reduced gametophytes. A basal branch of bryophytes in the plant phylogenetic tree would support the hypothesis that the dominant life cycle of gametophytes is plesiomorphic in embryophytes [78]. The proteins appeared to be conserved, and in a few cases seemed to diversify after the divergence of bryophytes and vascular plants, despite differences in spore/pollen development. The evolutionary history of the callose synthase subfamilies supports this hypothesis. CalS9 and CalS10 are clearly genetically linked, with both subfamilies highly supported (86% bootstrap support (BS), Fig 6), but CalS9 formed a second basal clade containing bryophyte (*Physcomitrella* and *Sphagnum*) and lycopod (*Selaginella*) callose synthases. CalS12 and CalS11, respectively, had a similar pattern. Moreover, CalS10 and CalS11 were not identified in any bryophyte or lycophyte, respectively, so they may have diverged by a duplication event in vascular plants after the divergence of bryophytes and lycopods. The results also supported the putative basal position of Selaginellales relative to all other vascular plants [78], because all members of the callose synthase subfamilies clustered together with bryophytes.

The only gymnosperm representative, *Pinus taeda*, contained the CalS13 protein. It was supported in the basal position of the CalS3 clade but only with low statistical support (in ML tree 69% BS and in MP tree 58% BS, see Figs 5 and 6). Pollen-wall structure and development differs greatly between gymnosperms and angiosperms [2]. Callose walls in gymnosperms form around the pollen mother cells and subsequently extend around each pollen microspore [2]. There, CalS13 becomes strongly expressed in the aperture region surrounding the emerging pollen tube after germination. In gymnosperms pollen tube cell wall does not contain callose and, accordingly, CalS13 does not extend into the pollen tube [19, 39].

The phylogenetic analyses identified six main highly supported clades containing all callose synthase subfamilies. However, group 2 and 5 were divided into two clades, first in ML analysis (S1 Fig) and the second in both, ML and MP (Figs 5 and 6). Clades 1, 2, and 6 in the trees (Figs 5 and 6) contained callose synthase subfamilies involved in late pollen development and pollen tube growth. Clade 1 contained CalS11 and CalS12 (100% BS), and clade 2 contained CalS9 and CalS10 (100% BS). CalS6 and CalS7 were in clade 3 (100% BS), CalS8 was in clade 4 (100% BS, Fig 5), and CalS1, CalS2, and CalS3 were in clade 5 (100% BS). Clade 6 contained CalS5 (100% BS, Fig 5). The roles of most of these subfamilies are not yet fully understood. CalS1 functions in cell-plate formation in sporophytic tissues [21], and CalS7 is required for the deposition of callose at the sieve plate [31]. All three clades formed distinct subclades with high support (99–100% BS, Fig 6). CalS6 and CalS4 may have evolved by duplication from CalS7 and CalS8, respectively. CalS2/1 in clade 5b may have evolved from CalS3 after the diversification of bryophyte CalS3 (clade 5a), which is also supported by the wider distribution of CalS3 than CalS2 and CalS1. In the light of CalS9/10 and CalS11/12 position in phylogenetic trees and their possible redundant function, we can agree with [11] that CalS1 and CalS2 are also possibly redundant. Observed high sequence homology also supports this statement.

Our data are in agreement with the report in [42] that most closely related CalS family members may have similar functions as a complex responsible for callose synthesis. We focused our attention mainly on five subfamilies of the 12 callose synthases that function during microsporogenesis and microgametogenesis (CalS5, CalS9, CalS10, CalS11 and CalS12). CalS9 and CalS10 were closely related within the CalS family. The CalS10 clade was embedded within CalS9 in the phylogenetic tree (97% BS in MP tree, Fig 6), supporting the suggestion in [42] that these members of the CalS family work as a complex. In [40], however, found that CalS10 and CalS9 were independently required for the development of male gametophytes and plant growth. In [79] reported similar results for cellulose synthase complexes that contained three cellulose synthase homologues. CalS11 and CalS12, which form a separate clade (95% BS in MP tree, Fig 6), are also genetically linked and have redundant roles in pollen development and fertility, especially in the formation of walls separating the microspores of the tetrad and late in the maturation of pollen [34].

The CalS5 subfamily formed a separate clade (and 100% in ML tree and 80% BS in MP tree, Figs 5 and 6), but its position was not clear. We hypothesized that all callose synthase subfamilies that play a role in the development of male gametophytes evolved separately from other CalS families. This hypothesis was supported by the ML analysis (Fig 5), where the clade expressing CalS5 in pollen was highly supported (100% BS). The monophyletic position of this group, however, was violated by bryophyte (*Marchantia polymorpha*, *Physcomitrella patens*, and *Sphagnum falax*) and lycopod (*Selaginella*) members of the CalS3 subfamily, which clustered together in clade 5a (Figs 5 and 6). Moreover, CalS5 in the more complete ML tree (S1 Fig) and rooted MP tree (Fig 6) was embedded in the subfamilies that were not expressed in mature pollen but were expressed in early stages of male gametophyte development instead (CalS1, CalS2, CalS3 and CalS4, clade 5). Some positions of CalS5 in basal angiosperms remained problematic, because only partial sequences were available (but formed a separate clade with unresolved positions from each other).

Interestingly, most callose synthases expressed in *A*. *thaliana* sperm cells were phylogenetically related to CalS5, forming clades 4 a 5 (CalS1, CalS2, CalS3 and CalS8), although none of them was apparently pollen-specific. The only exception was CalS10 which also showed expression signal in sperm cells but its expression was much lower than that of the other sperm cell-expressed genes. It would be interesting to investigate whether the sequence diversification between clades 5 (CalS1/2/3) and 6 (CalS5), that was also highlighted by the expression profiles of their members, reflects only regulatory or also functional specialization.

Our analyses led to the description of highly conserved Vta1-FKS1-GluSynt module (Fig 7) of callose synthases in all subfamilies across the plant kingdom. The N-terminal Vta1 domain however, was sometimes missing in CalS9 and CalS10. This division was fully consistent with a close relationship between these two subfamilies (Figs 5 and 6). In CalS9, the Vta1 was missing in most of the bryophytes, except for *M*. *polymorpha*, basal angiosperms, and monocots. Vta1 in CalS9 was also absent in dicots, except for the genera *Arabidopsis*, *Brassica* and *Eutrema*. Vta1 in CalS10 was absent from most of the plant species, except for the monocot *Zostera marina* (Alismatales) and the dicots *Medicago truncatula* (Fabales) and *Nicotiana alata* (Solanales). These findings suggested the convergence amongst individual plant species. In contrast, the evolution of other regions of the proteins was more dynamic. The comparative analysis of the CalS proteins identified 81 types of protein domains (Fig 7). Seven subfamilies shared more variable C termini (CalS4, 5, 6, 7, 10, 11, and 12), but others varied across the entire region (CalS1, 3, 8, and 9). Bryophytes and basal angiosperms more often contained motifs not shared with other plants, in agreement with their phylogenetic positions.

The 12 callose synthase genes can be divided into two groups based on gene structure in *Arabidopsis thaliana* [26]. The first group harbours CalS11 and Cals12 genes which have only two or three exons and the second group contains from 40 to 50 exons in CalS1- CalS10 (Fig 8). Nevertheless, this composition is not same in all plant species as we shown in basal angiosperm *Amborella trichopoda*, where e.g. CalS12 contains only one exon and CalS3 43 exons. We can point out that the structural divergence among different callose synthases within plant kingdom is generally high and clearly corresponds with their phylogenetic positions of studied species.

## Supporting information

S1 Table Domain sequence distribution in different callose synthases from the Pfam Motif Library. Highlighted domains are found in different plants.
(XLSX)

S2 Table Consensus-sequence blocks of plant callose synthases. Sequence logos indicates main 50 conserved sequence patterns.
(DOCX)

S1 Fig Phylogenetic tree and classification of 1211 plant sequences of 12 *Arabidopsis* callose synthase proteins. The evolutionary history was inferred by using maximum likelihood; the tree with the highest log likelihood (-979085.0765) is shown. The analysis included a total of 4150 positions in the final data set. Main branches with circles are described in the text.
(TIF)

S2 Fig Phylogenetic tree of the pollen-specific CalS5 (GLS2) protein rooted with *Ginkgo biloba*. The timetree shown was generated using RelTime. The estimated log likelihood value of the topology shown is -57276.0114. A discrete gamma distribution was used to model the differences in evolutionary rate amongst sites (five categories (+*G*, parameter = 0.5505)). The model of rate variation allowed some sites to be evolutionarily invariable ([+*I*], 11.2557% sites). The tree is drawn to scale, with branch lengths measured in relative number of substitutions per site. The analysis included 105 amino acid sequences, and the final data set had a total of 2190 positions.
(TIF)

S3 Fig Phylogenetic tree of pollen-expressed CalS9 (GLS10) proteins rooted with *Physcomitrella patens*. The timetree shown was generated using RelTime. The estimated log likelihood value of the topology shown is -91929.9006. A discrete gamma distribution was used to model the differences in evolutionary rate amongst sites (five categories (+*G*, parameter = 0.8543)). The model of rate variation allowed some sites to be evolutionarily invariable ([+*I*], 7.5727% sites). The tree is drawn to scale, with branch lengths measured in relative number of substitutions per site. The analysis included 127 amino acid sequences, and the final data set had a total of 2476 positions.
(TIF)

S4 Fig Phylogenetic tree of pollen-expressed CalS10 (GLS8) proteins rooted with *Amborella trichopoda*. The timetree shown was generated using RelTime. The estimated log likelihood value of the topology shown is -54519.4579. A discrete gamma distribution was used to model the differences in evolutionary rate amongst sites five categories (+*G*, parameter = 0.5253)). The model of rate variation allowed some sites to be evolutionarily invariable ([+*I*], 0.7256% sites). The tree is drawn to scale, with branch lengths measured in relative number of substitutions per site. The analysis included 104 amino acid sequences, and the final data set had a total of 2274 positions.
(TIF)

S5 Fig Phylogenetic tree of pollen-expressed CalS11 (GLS1) proteins rooted with *Spirodella polyrhiza*. The timetree shown was generated using RelTime. The estimated log likelihood value of the topology shown is -54506.2366. A discrete gamma distribution was used to model the differences in evolutionary rate amongst sites (five categories (+*G*, parameter = 0.5988)). The model of rate variation allowed some sites to be evolutionarily invariable ([+*I*], 12.0845% sites). The tree is drawn to scale, with branch lengths measured in relative number of substitutions per site. The analysis included 84 amino acid sequences, and the final data set had a total of 2106 positions.
(TIF)

S6 Fig Phylogenetic tree of pollen-expressed CalS 12 (GLS5) proteins rooted with *Physcomitrella patens*. The timetree shown was generated using RelTime. The estimated log likelihood value of the topology shown is -79253.3574. A discrete gamma distribution was used to model the differences in evolutionary rate amongst sites (five categories (+*G*, parameter = 0.6942)). The model of rate variation allowed some sites to be evolutionarily invariable ([+*I*], 13.5059% sites). The tree is drawn to scale, with branch lengths measured in relative number of substitutions per site. The analysis included 139 amino acid sequences, and the final data set had a total of 2299 positions.
(TIF)

S1 Nexus File Callose synthase amino acid alignment data set. (NEX)
