# Supplementary material for: Evolutionary history of callose synthases in terrestrial plants with emphasis on proteins involved in male gametophyte development
Source: PLoS One. 2017 Nov 13;12(11):e0187331. doi: 10.1371/journal.pone.0187331 (PMC5683620; doi:10.1371/journal.pone.0187331)
Supplement: S2 Table — Sequence logos indicates main 50 conserved sequence patterns. (DOCX) [file pone.0187331.s002.docx]

| **Block no.** | **Length (bp)** | **Consensus sequence** | **Domain component** | **Specific CalS protein** |
| --- | --- | --- | --- | --- |
| 1 | 41 | 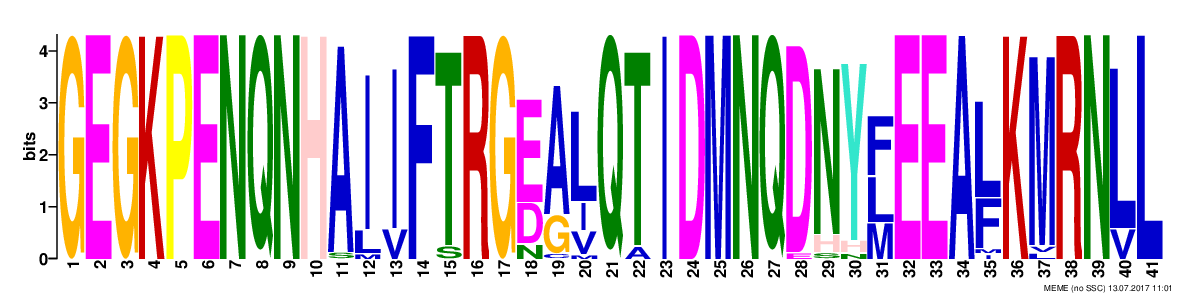 | Glucan synthase | all |
| 2 | 41 | 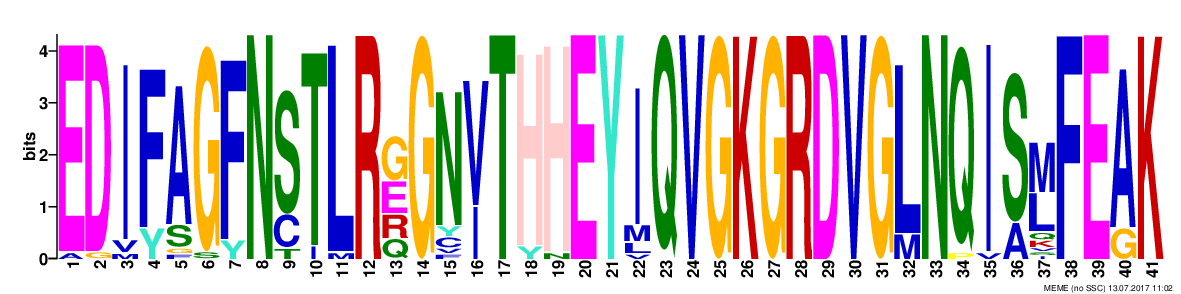 | Glucan synthase | all |
| 3 | 39 | 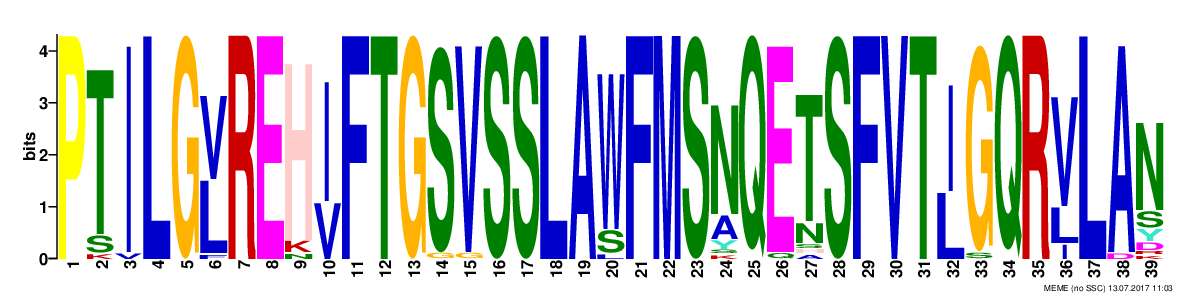 | Glucan synthase | all |
| 4 | 50 | 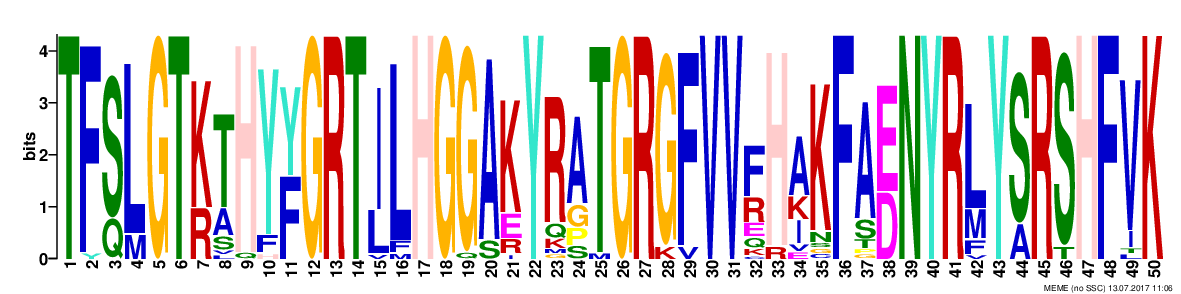 | Glucan synthase | all |
| 5 | 50 | 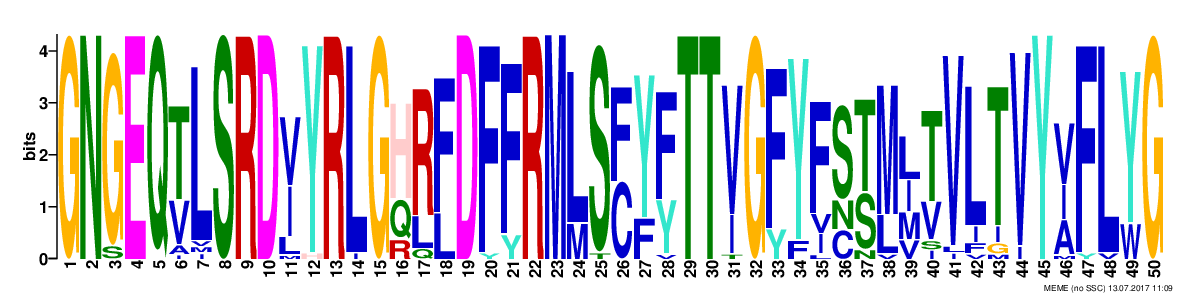 | Glucan synthase | all |
| 6 | 50 | 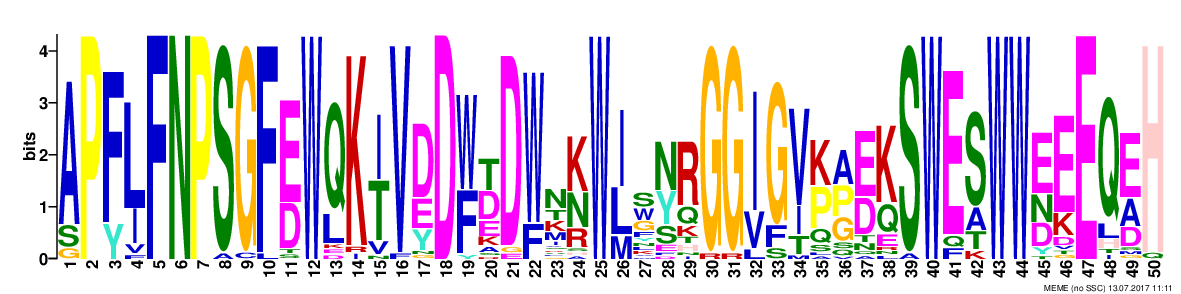 | Glucan synthase | all |
| 7 | 50 | 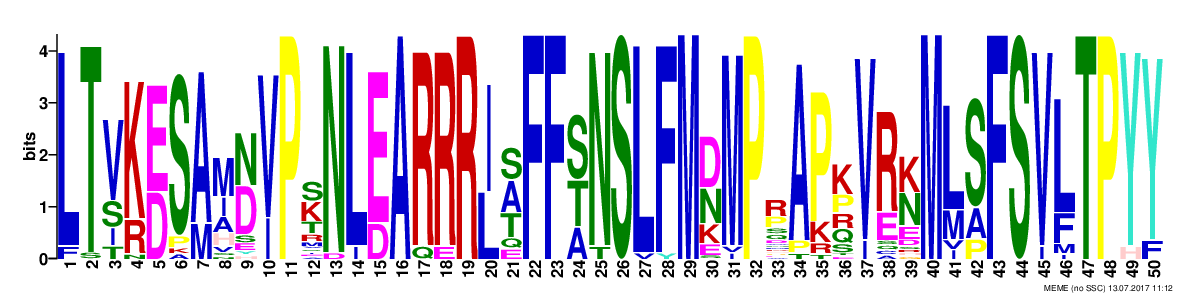 | Glucan synthase | all |
| 8 | 50 | 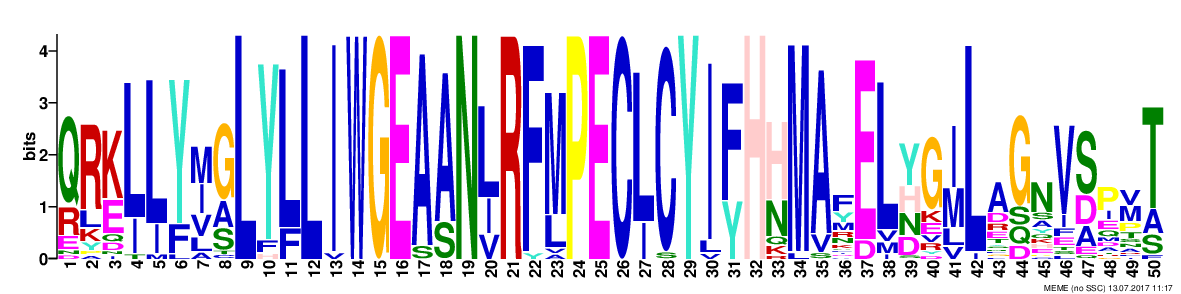 | FKS1 | all |
| 9 | 41 | 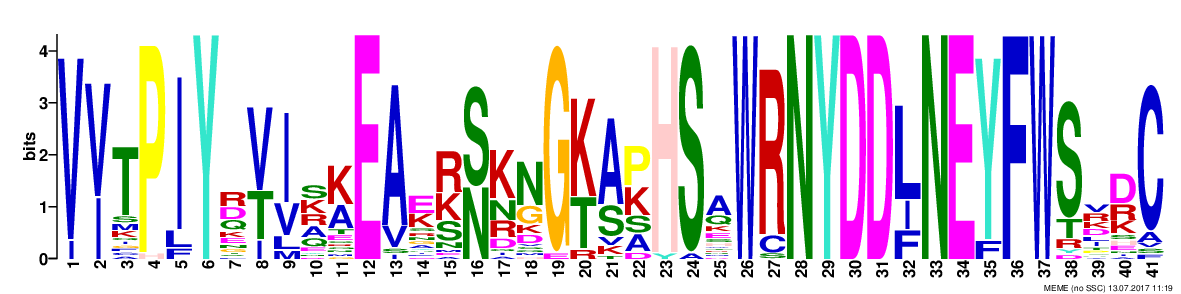 | FKS1 | all |
| 10 | 29 | 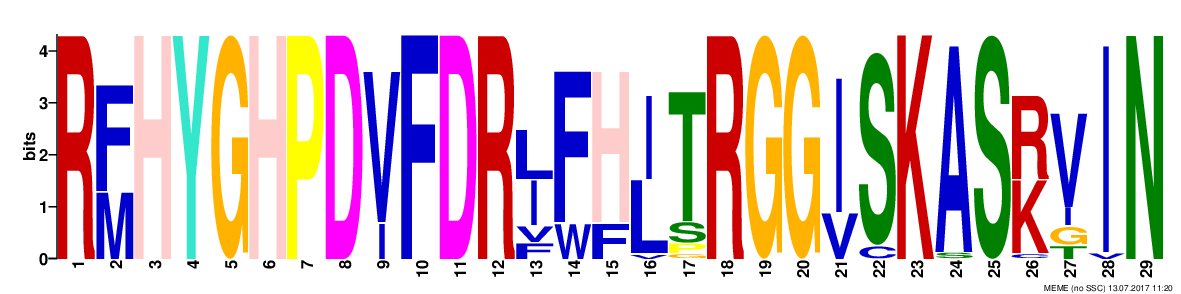 | Glucan synthase | all |
| 11 | 36 | 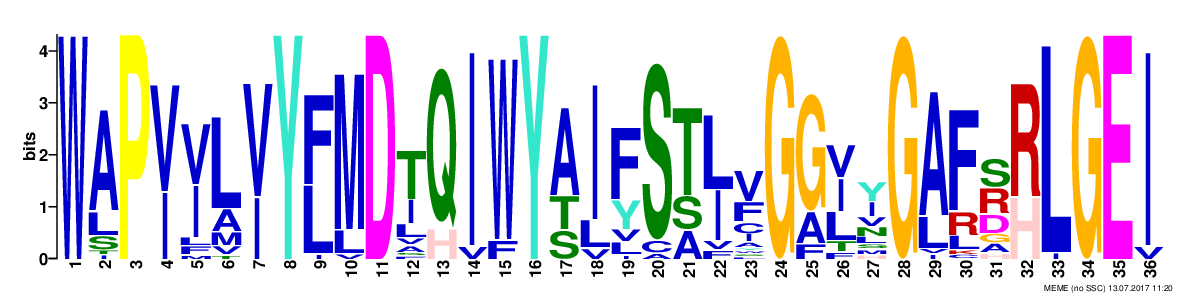 | Transmembrane domain | all |
| 12 | 50 | 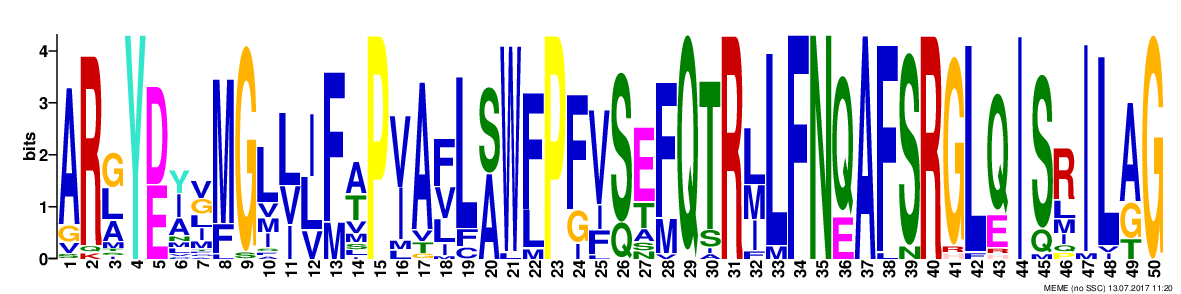 | Transmembrane domain | all |
| 13 | 50 | 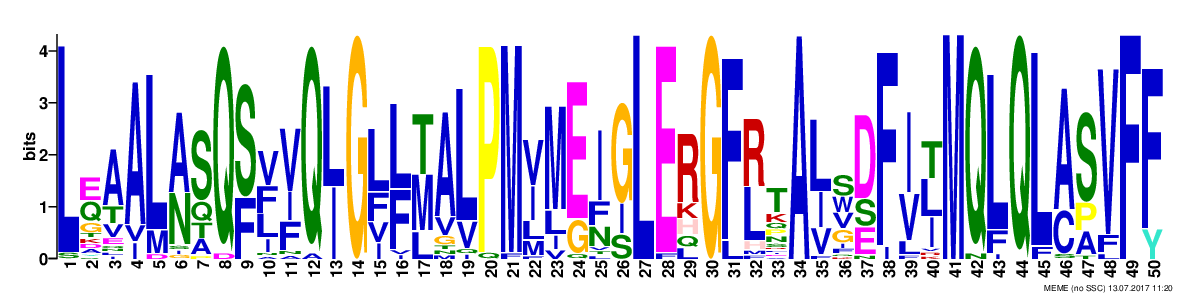 | Glucan synthase | all |
| 14 | 30 | 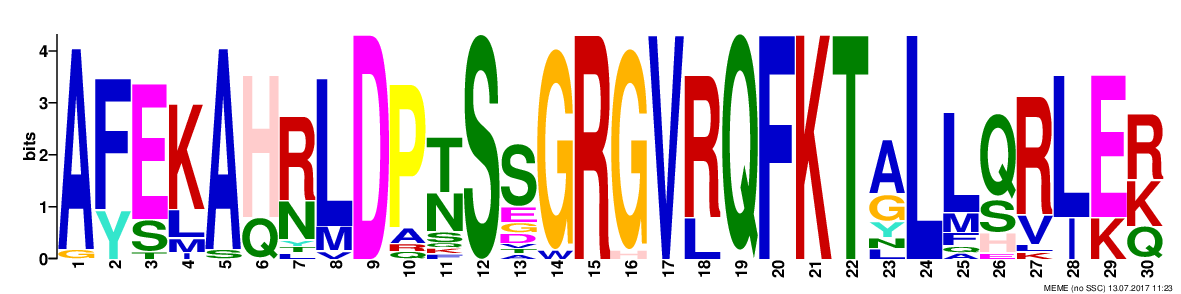 | - | CalS 1, 2, 3, 4, 5, 6, 7, 9, 10 |
| 15 | 41 | 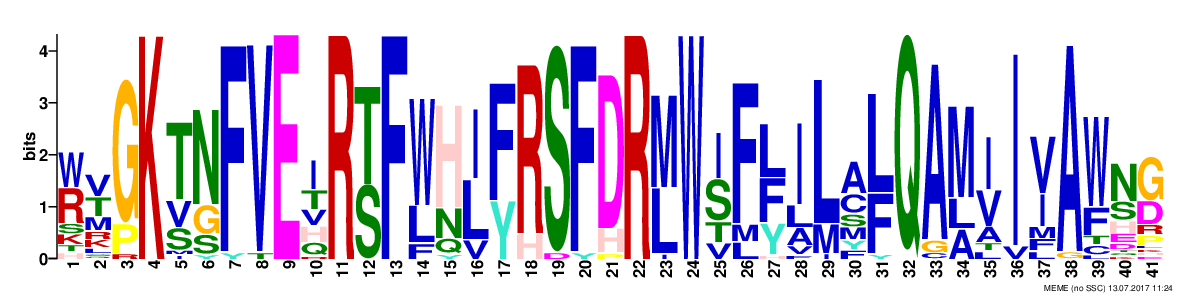 | Transmembrane domain | all |
| 16 | 25 | 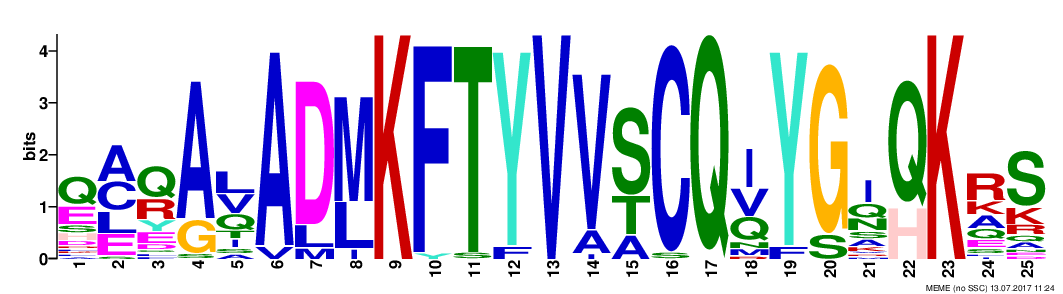 | - | all |
| 17 | 50 | 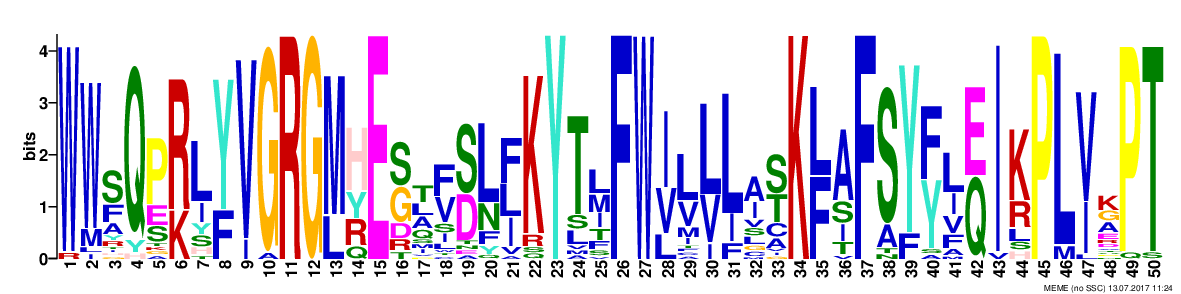 | Transmembrane domain | all |
| 18 | 28 | 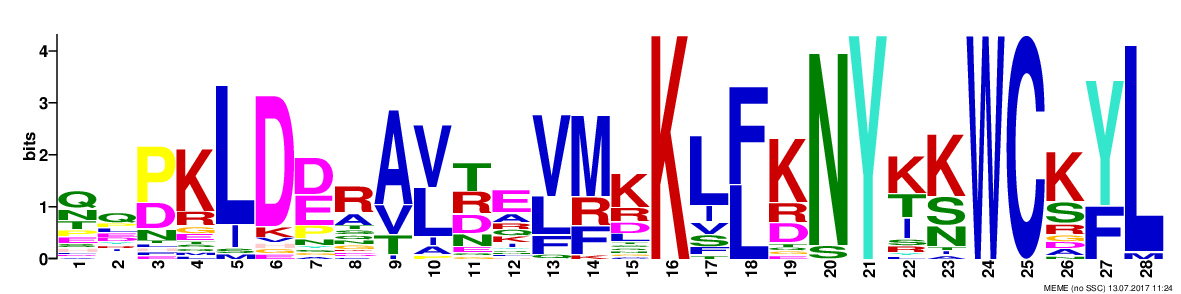 | - | all |
| 19 | 20 | 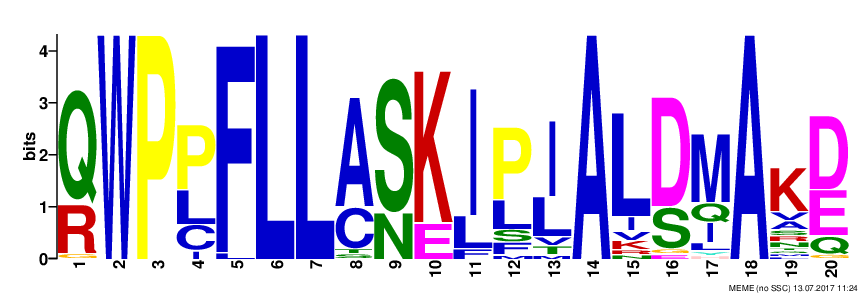 | - | all |
| 20 | 29 | 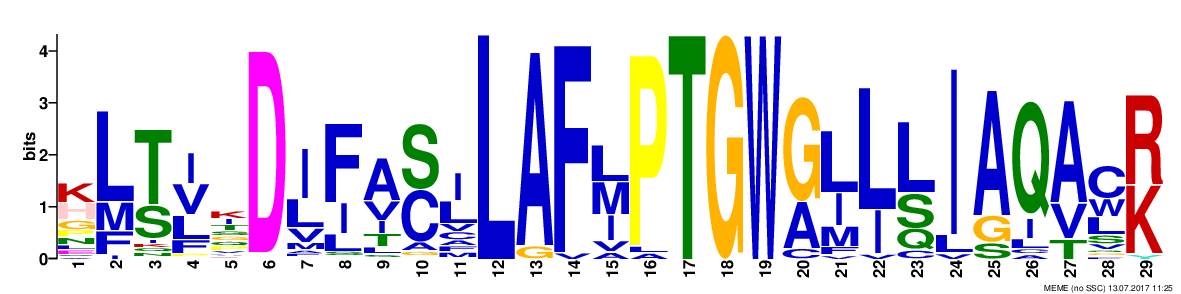 | - | all |
| 21 | 29 | 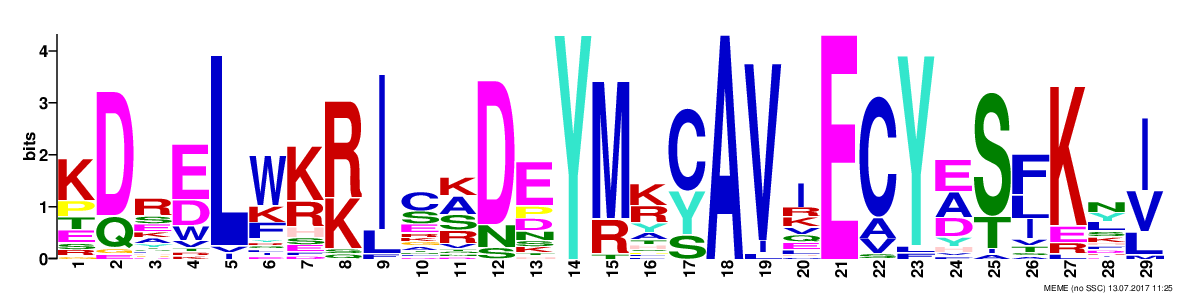 | - | all |
| 22 | 14 | 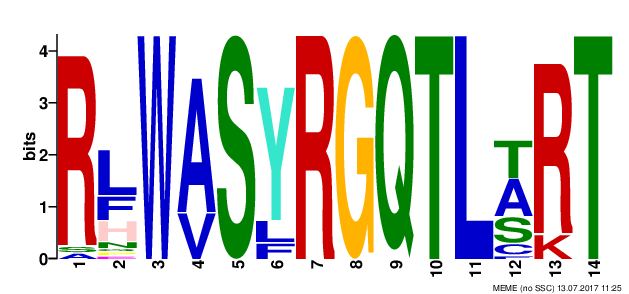 | - | all |
| 23 | 50 | 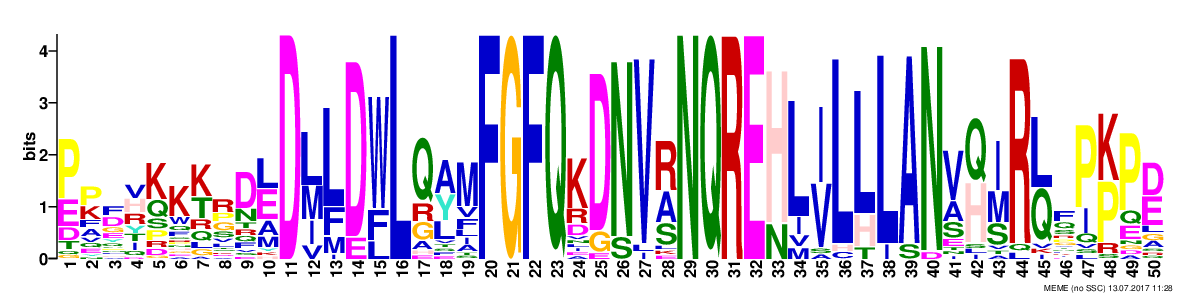 | - | CalS 1, 2, 3, 4, 5, 7, 9, 10, 11, 12 |
| 24 | 29 | 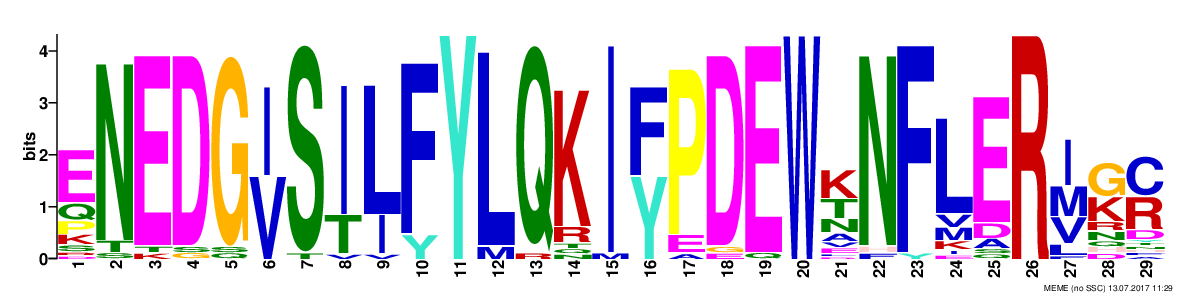 | - | all |
| 25 | 36 | 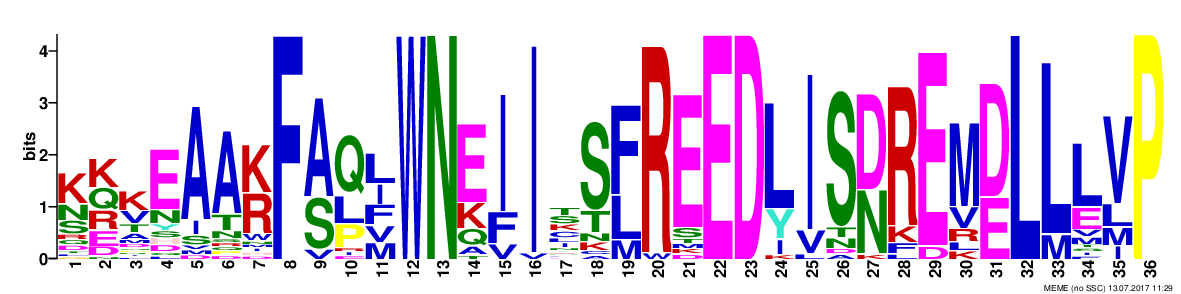 | - | all |
| 26 | 29 | 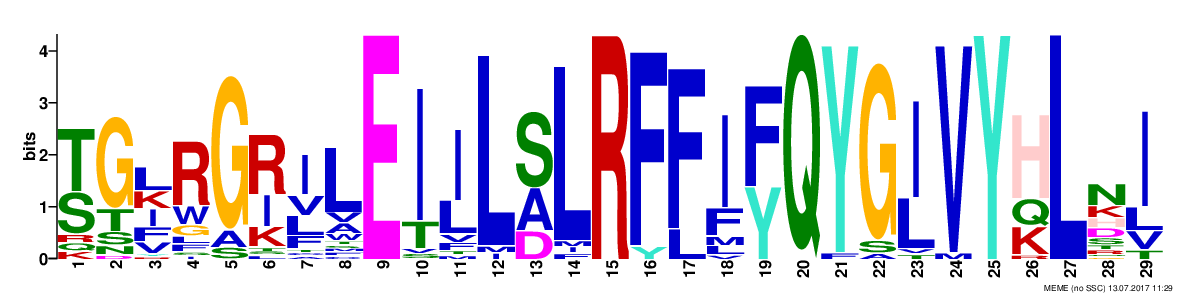 | - | all |
| 27 | 29 | 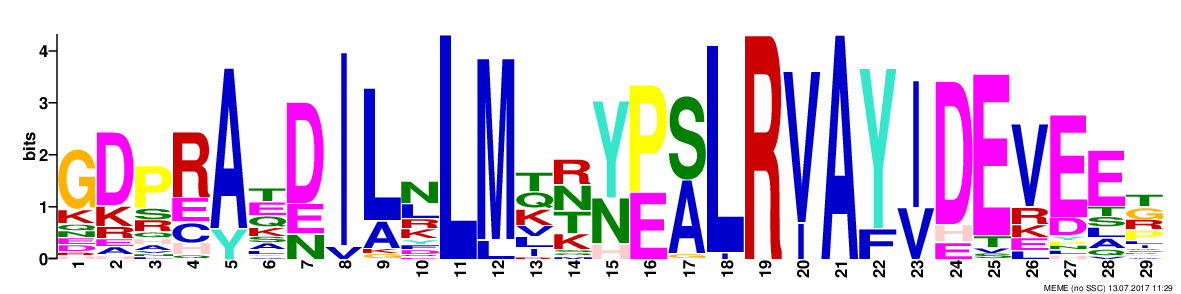 | - | all |
| 28 | 29 | 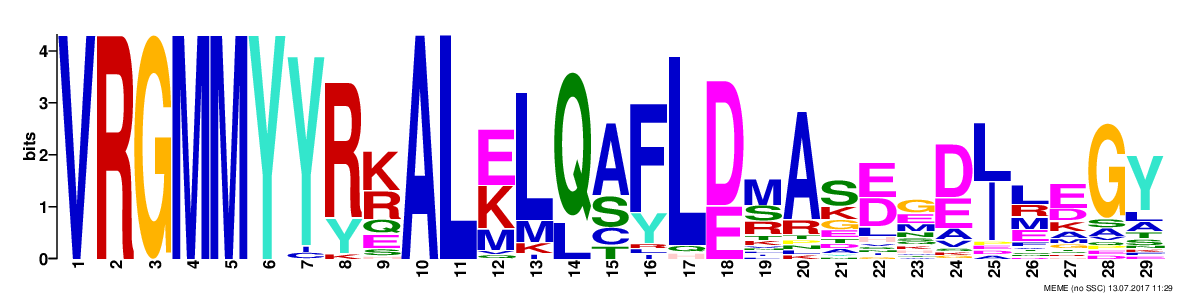 | - | all |
| 29 | 33 | 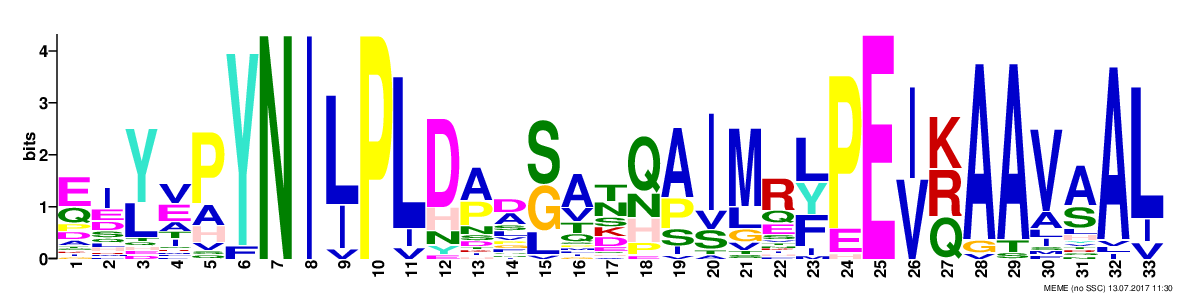 | - | all |
| 30 | 29 | 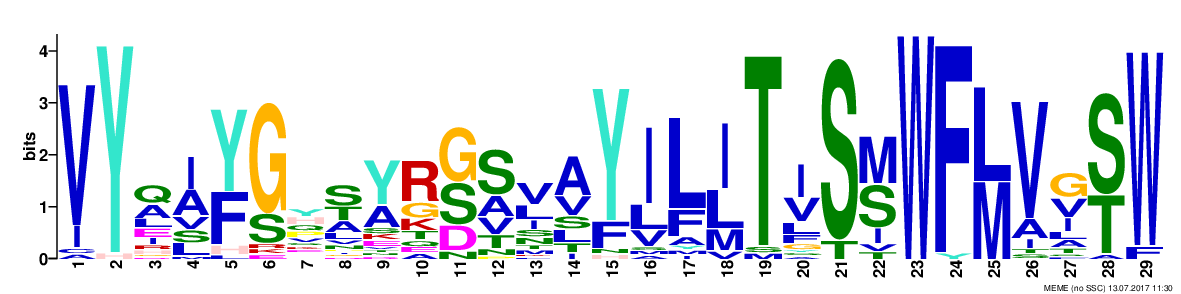 | - | all |
| 31 | 25 | 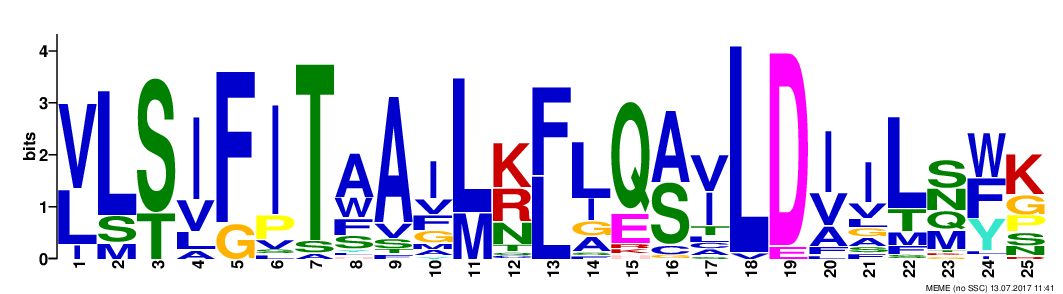 | - | all |
| 32 | 25 | 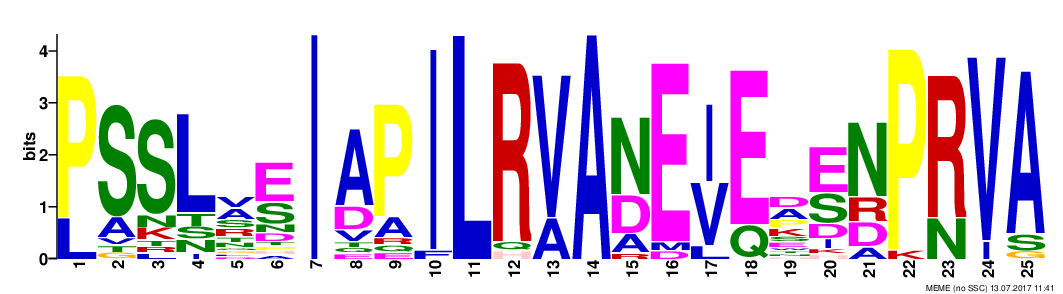 | - | CalS 1, 2, 3, 4, 5, 7, 9 |
| 33 | 30 | 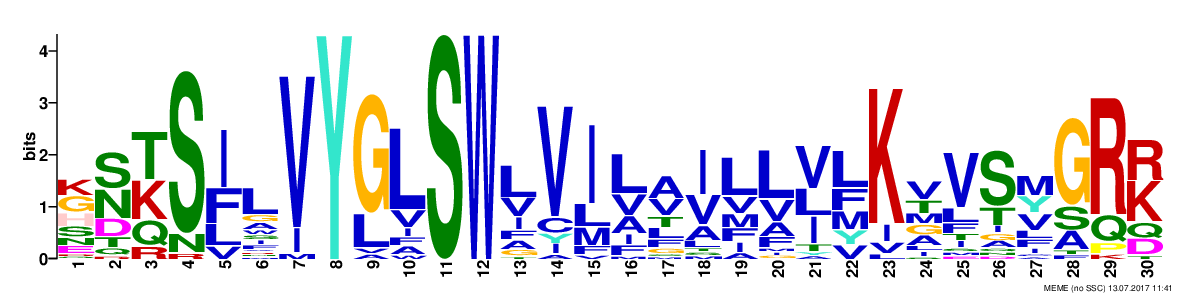 | - | all |
| 34 | 21 | 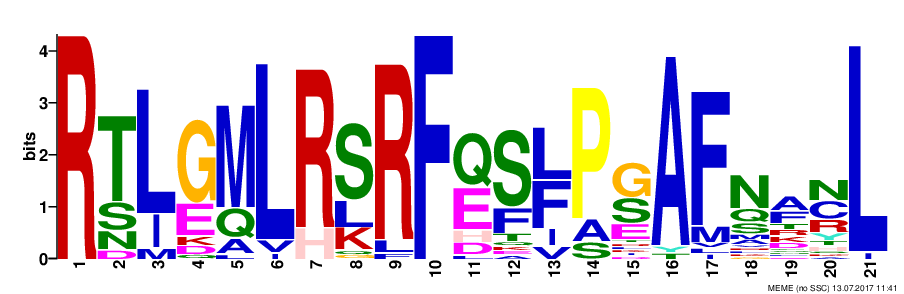 | - | all |
| 35 | 41 | 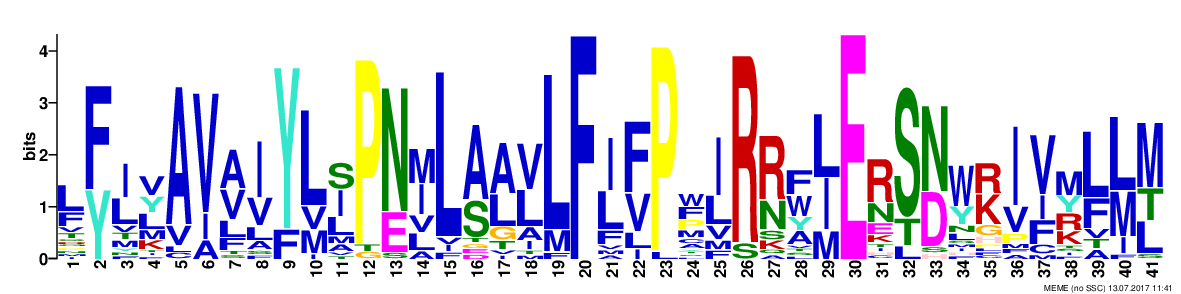 | Transmembrane domain | all |
| 36 | 39 | 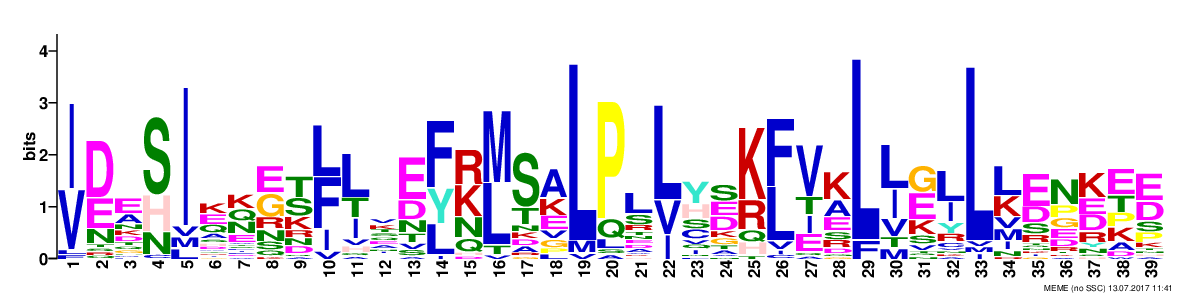 | - | all |
| 37 | 15 | 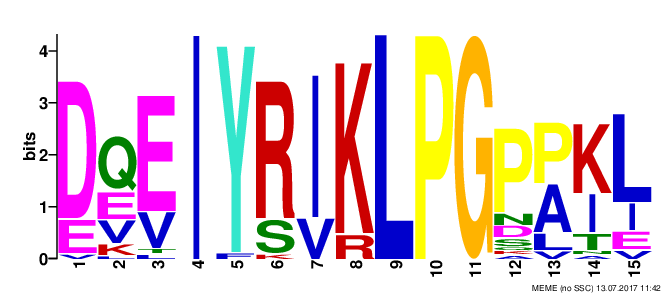 | - | all |
| 38 | 29 | 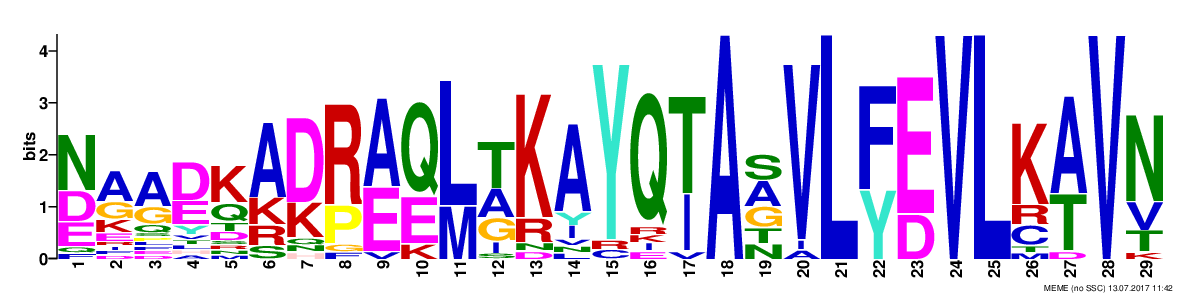 | - | all |
| 39 | 21 | 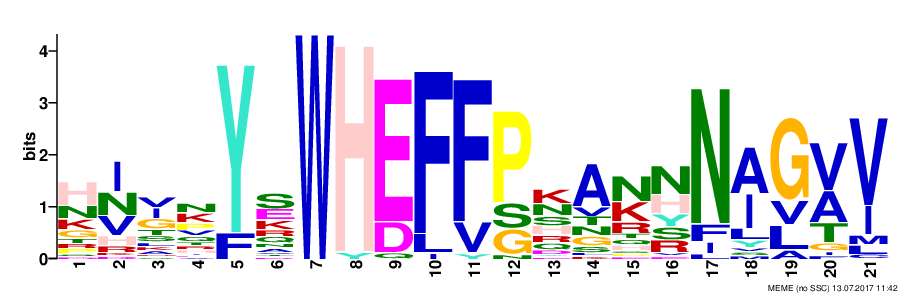 | - | all |
| 40 | 29 | 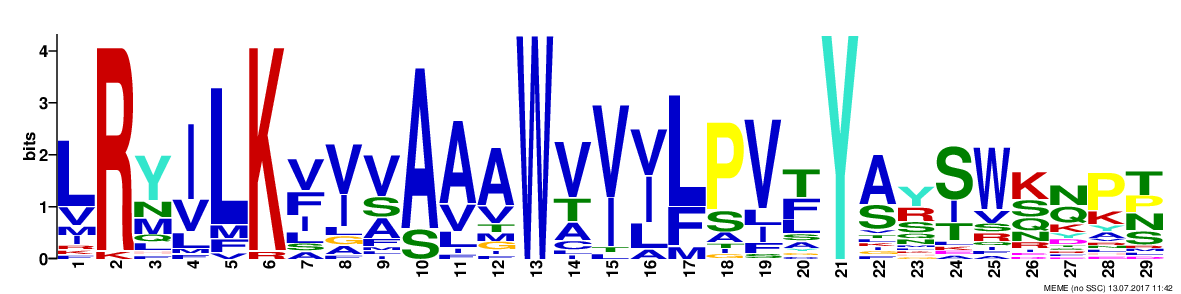 | - | all |
| 41 | 21 | 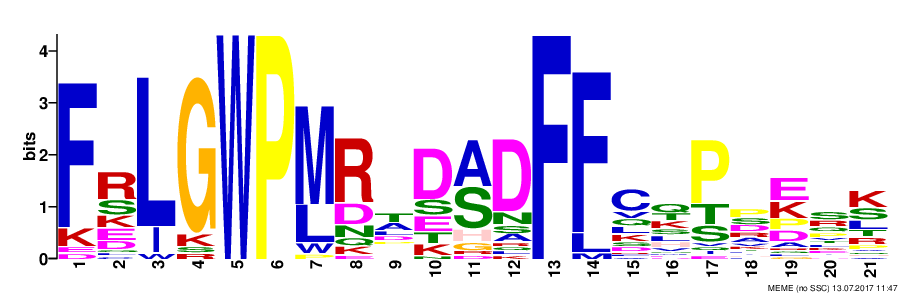 | - | all |
| 42 | 29 | 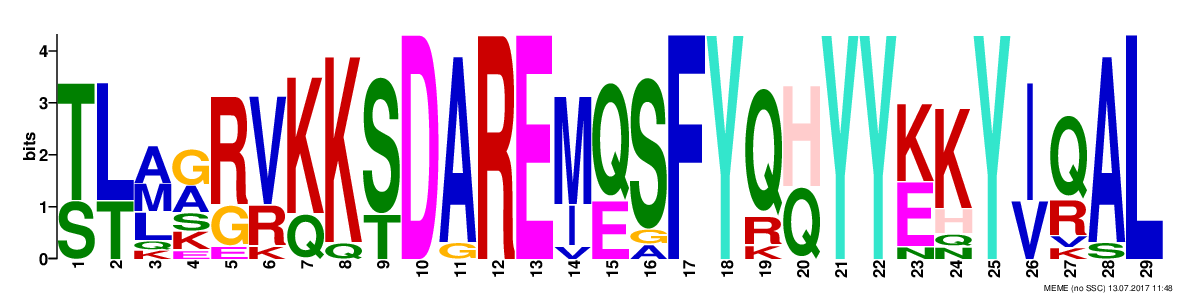 | - | CalS 1, 2, 3, 5 |
| 43 | 21 | 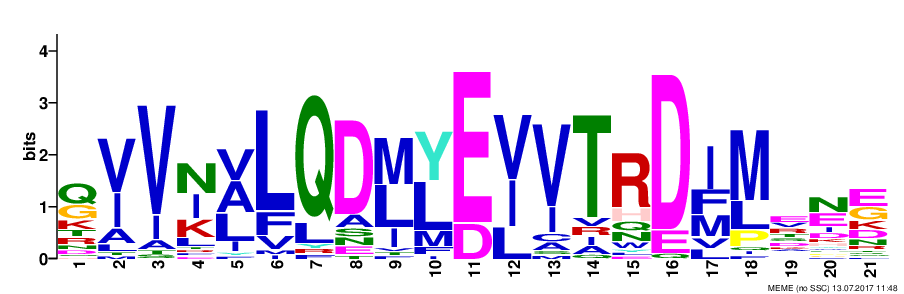 | - | all |
| 44 | 21 | 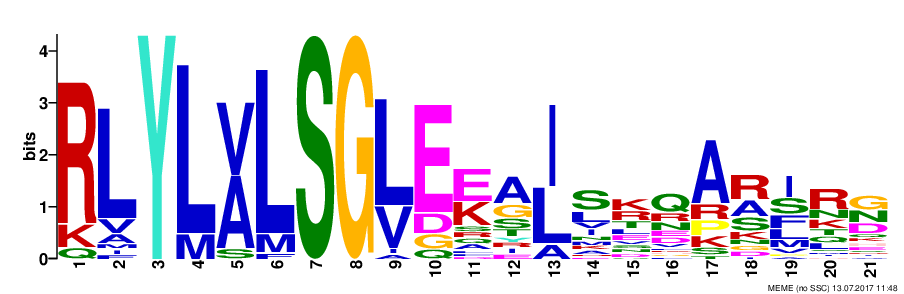 | - | all |
| 45 | 21 | 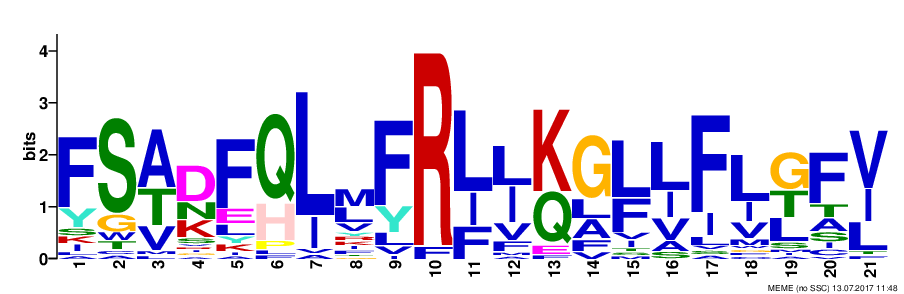 | - | all |
| 46 | 15 | 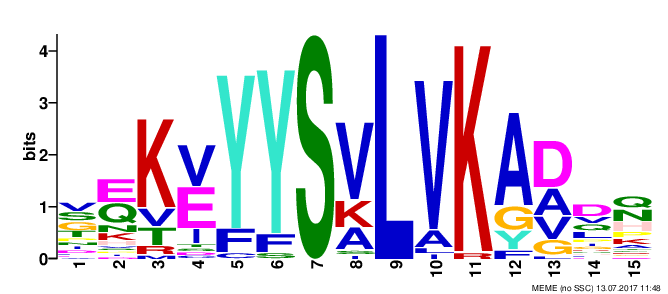 | - | all |
| 47 | 15 | 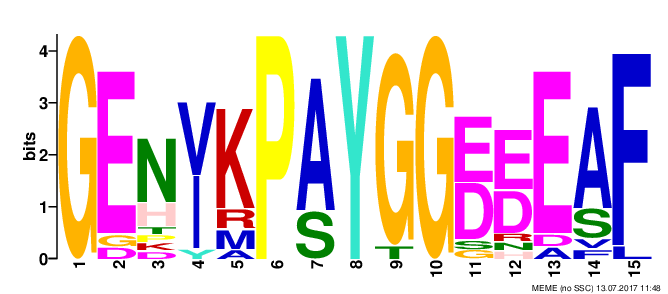 | - | CalS 1, 2, 3, 4, 5, 7, 8, 9 |
| 48 | 15 | 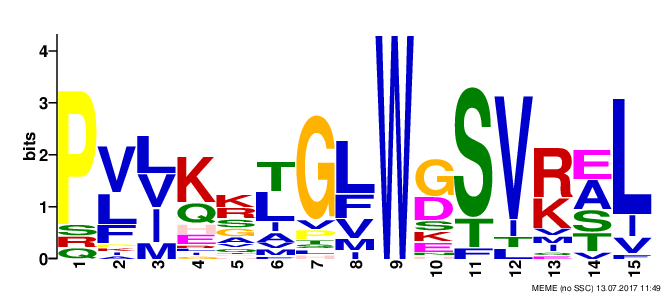 | - | all |
| 49 | 15 | 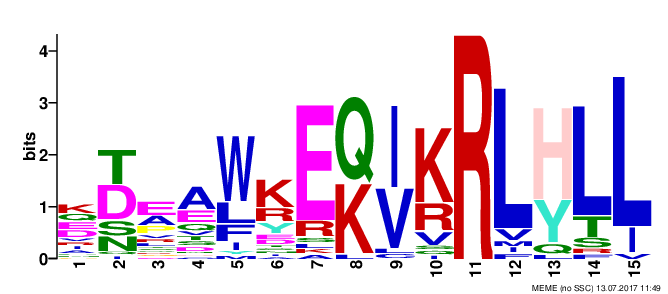 | - | all |
| 50 | 49 | 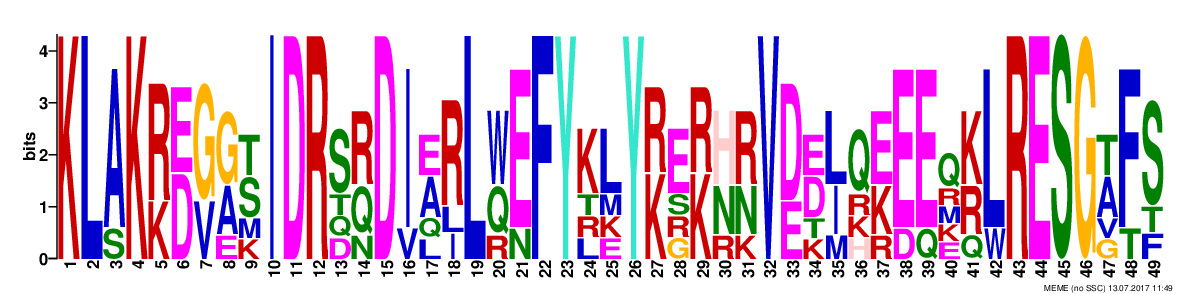 | - | CalS 9, 10 |
