# Supplementary figures and images for: Evolutionary history of callose synthases in terrestrial plants with emphasis on proteins involved in male gametophyte development
Source: PLoS One. 2017 Nov 13;12(11):e0187331. doi: 10.1371/journal.pone.0187331 (PMC5683620; doi:10.1371/journal.pone.0187331)

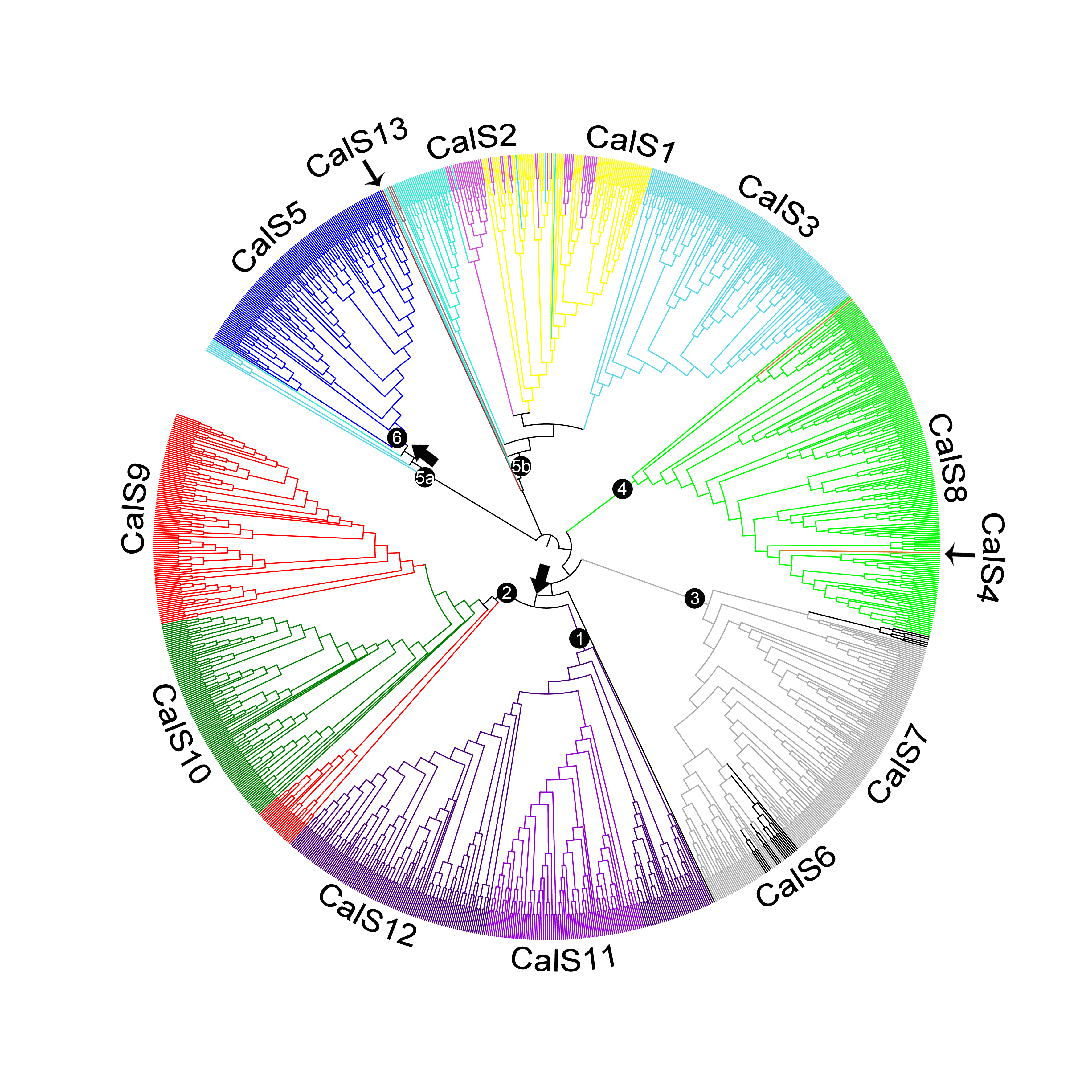

Supplement: S1 Fig — The evolutionary history was inferred by using maximum likelihood; the tree with the highest log likelihood (-979085.0765) is shown. The analysis included a total of 4150 positions in the final data set. Main branches with circles are described in the text. (TIF) [file pone.0187331.s003.tif]

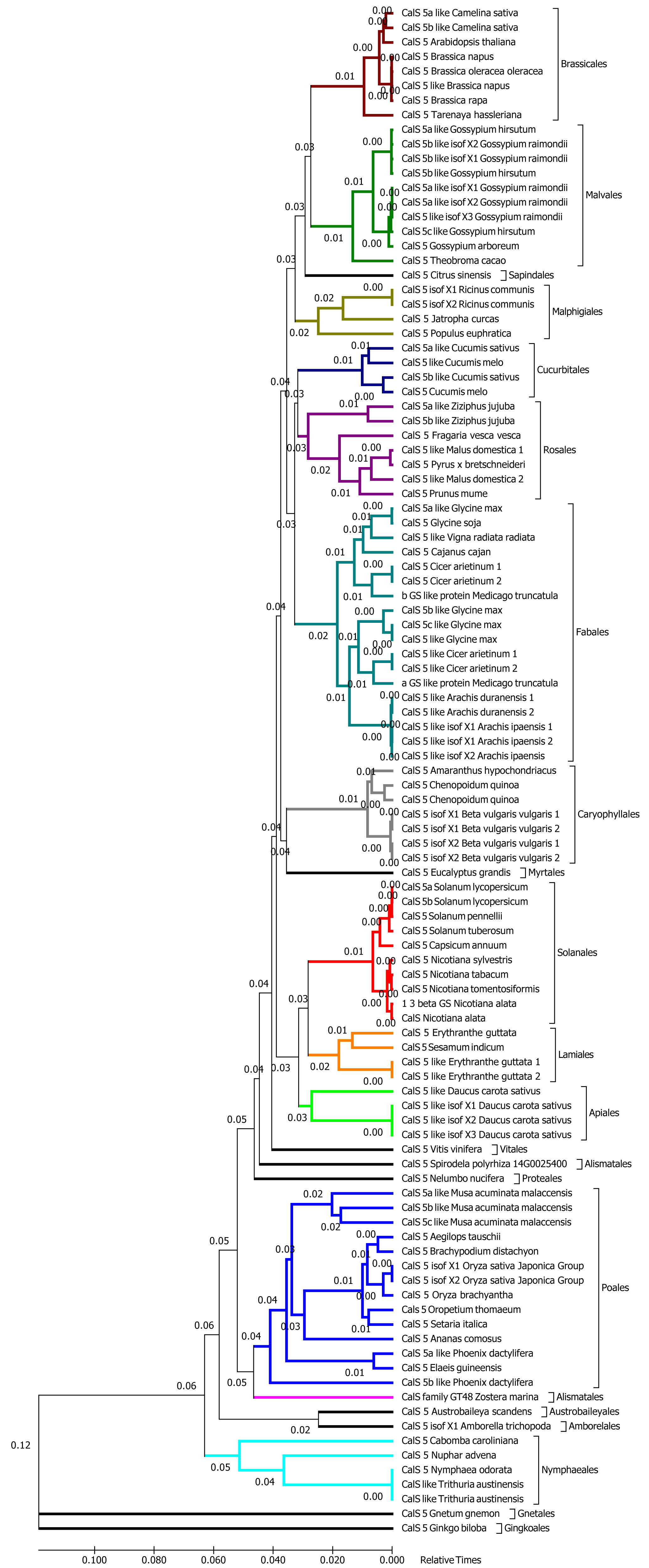

Supplement: S2 Fig — The timetree shown was generated using RelTime. The estimated log likelihood value of the topology shown is -57276.0114. A discrete gamma distribution was used to model the differences in evolutionary rate amongst sites (five categories (+G, parameter = 0.5505)). The model of rate variation allowed some sites to be evolutionarily invariable ([+I], 11.2557% sites). The tree is drawn to scale, with branch lengths measured in relative number of substitutions per site. The analysis included 105 amino acid sequences, and the final data set had a total of 2190 positions. (TIF) [file pone.0187331.s004.tif]

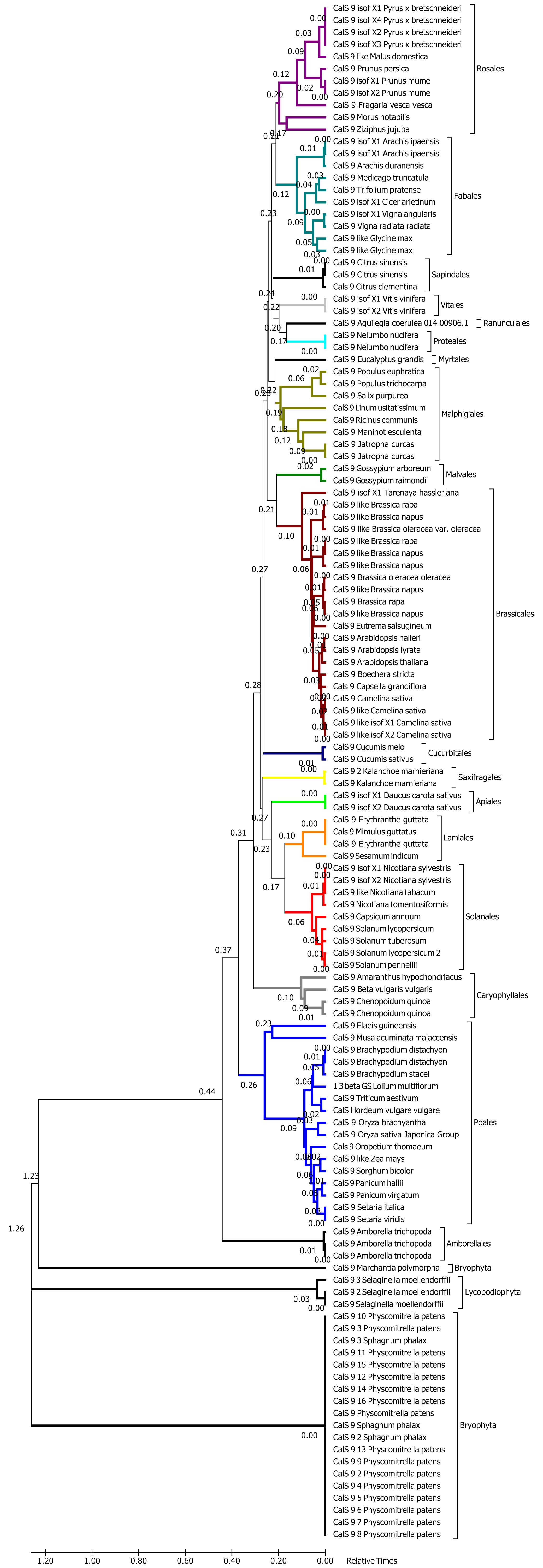

Supplement: S3 Fig — The timetree shown was generated using RelTime. The estimated log likelihood value of the topology shown is -91929.9006. A discrete gamma distribution was used to model the differences in evolutionary rate amongst sites (five categories (+G, parameter = 0.8543)). The model of rate variation allowed some sites to be evolutionarily invariable ([+I], 7.5727% sites). The tree is drawn to scale, with branch lengths measured in relative number of substitutions per site. The analysis included 127 amino acid sequences, and the final data set had a total of 2476 positions. (TIF) [file pone.0187331.s005.tif]

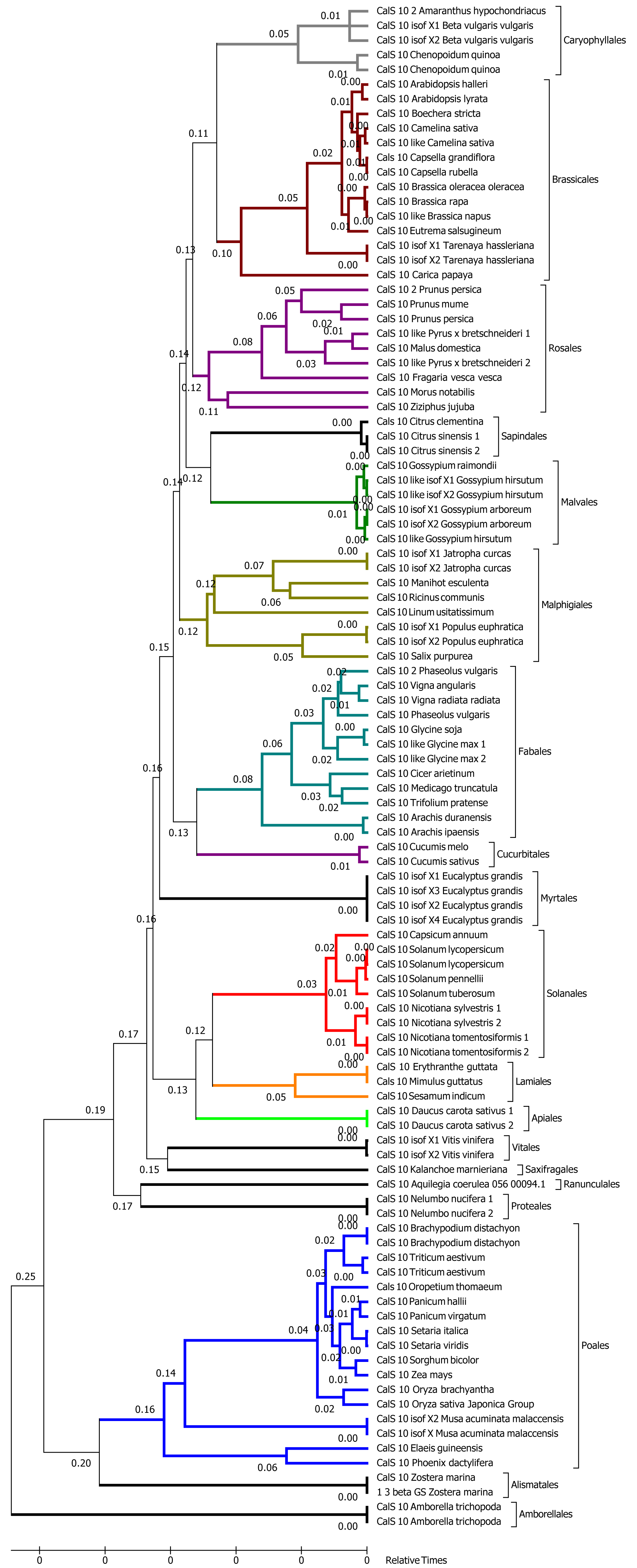

Supplement: S4 Fig — The timetree shown was generated using RelTime. The estimated log likelihood value of the topology shown is -54519.4579. A discrete gamma distribution was used to model the differences in evolutionary rate amongst sites five categories (+G, parameter = 0.5253)). The model of rate variation allowed some sites to be evolutionarily invariable ([+I], 0.7256% sites). The tree is drawn to scale, with branch lengths measured in relative number of substitutions per site. The analysis included 104 amino acid sequences, and the final data set had a total of 2274 positions. (TIF) [file pone.0187331.s006.tif]

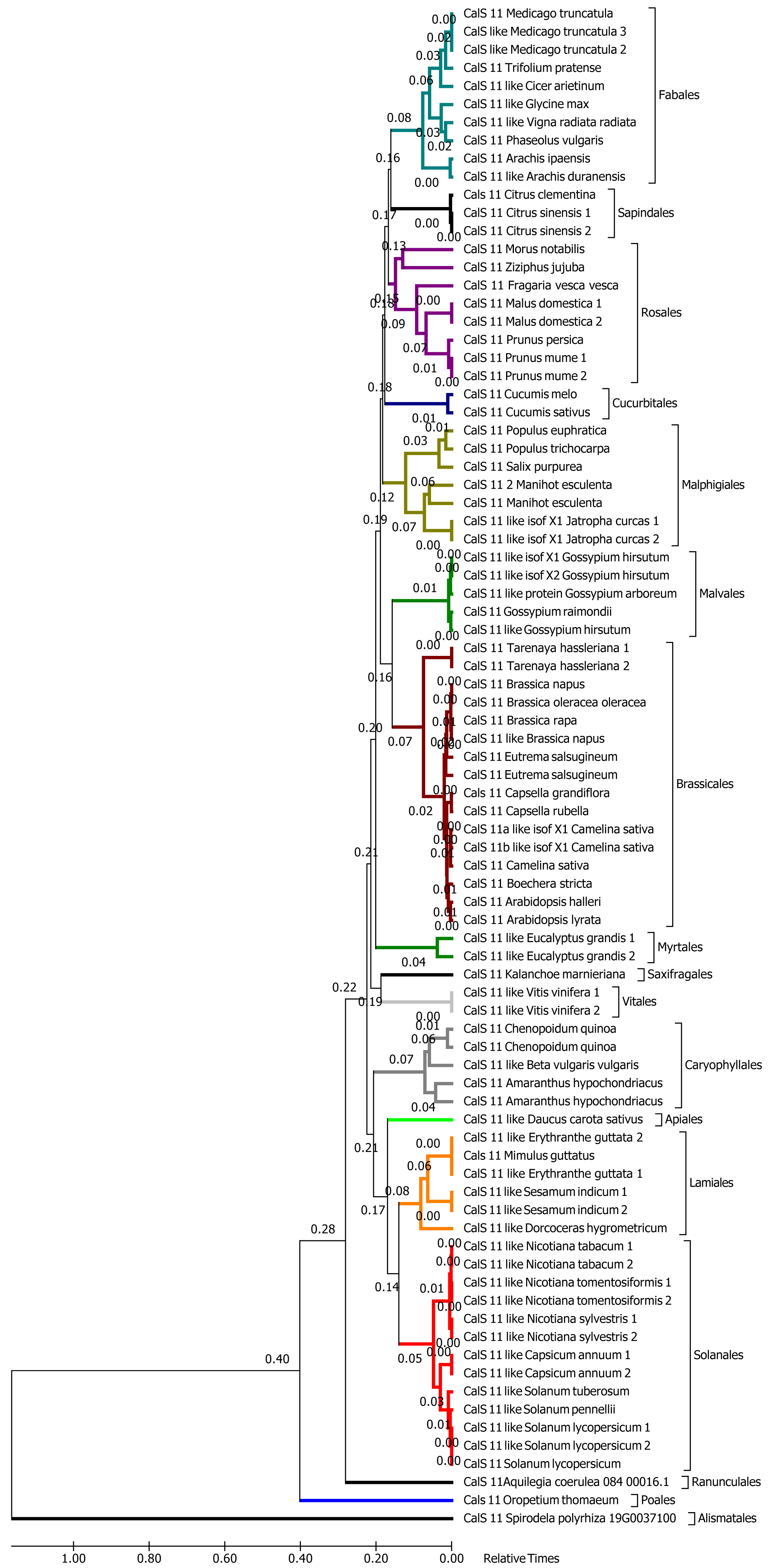

Supplement: S5 Fig — The timetree shown was generated using RelTime. The estimated log likelihood value of the topology shown is -54506.2366. A discrete gamma distribution was used to model the differences in evolutionary rate amongst sites (five categories (+G, parameter = 0.5988)). The model of rate variation allowed some sites to be evolutionarily invariable ([+I], 12.0845% sites). The tree is drawn to scale, with branch lengths measured in relative number of substitutions per site. The analysis included 84 amino acid sequences, and the final data set had a total of 2106 positions. (TIF) [file pone.0187331.s007.tif]

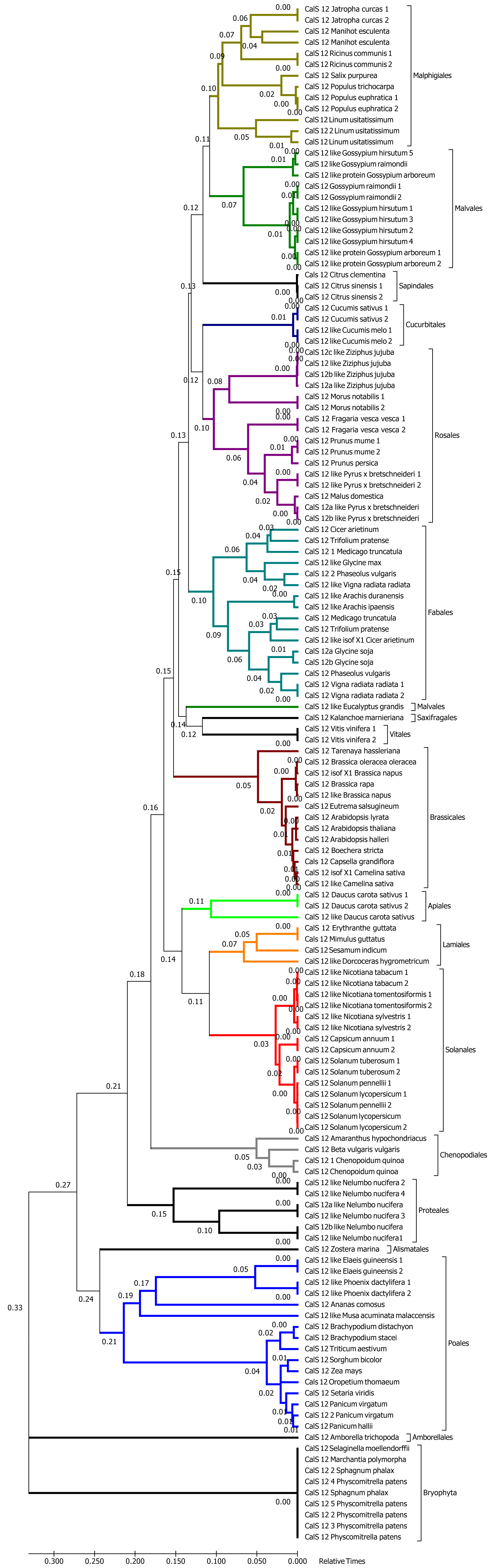

Supplement: S6 Fig — The timetree shown was generated using RelTime. The estimated log likelihood value of the topology shown is -79253.3574. A discrete gamma distribution was used to model the differences in evolutionary rate amongst sites (five categories (+G, parameter = 0.6942)). The model of rate variation allowed some sites to be evolutionarily invariable ([+I], 13.5059% sites). The tree is drawn to scale, with branch lengths measured in relative number of substitutions per site. The analysis included 139 amino acid sequences, and the final data set had a total of 2299 positions. (TIF) [file pone.0187331.s008.tif]
